# Global transcriptome analysis uncovers the gene co-expression regulation network and key genes involved in grain development of wheat (*Triticum aestivum* L.)

**DOI:** 10.1007/s10142-019-00678-z

**Published:** 2019-05-21

**Authors:** Qing Chi, Lijian Guo, Meng Ma, Lijian Zhang, Hude Mao, Baowei Wu, Xiangli Liu, Ricardo H. Ramirez-Gonzalez, Cristobal Uauy, Rudi Appels, Huixian Zhao

**Affiliations:** 1 College of Life Sciences, Northwest A & F University, Yangling, 712100 Shaanxi China; 2 College of Plant Protection, Northwest A & F University, Yangling, 712100 Shaanxi China; 3 State Key Laboratory of Crop Stress Biology for Arid Areas, Northwest A & F University, Yangling, 712100 Shaanxi China; 4 John Innes Centre, Norwich Research Park, Norwich, NR4 7UH UK; 5 School of BioSciences, University of Melbourne, Parkville, VIC 3010 Australia

**Keywords:** Wheat, Grain development, Transcriptome, Differentially expressed genes, Transcription factors, Gene co-expression regulation network

## Abstract

Wheat grain development is a robust biological process that largely determines grain quality and yield. In this study, we investigated the grain transcriptome of winter wheat cv. Xiaoyan-6 at four developmental stages (5, 10, 15, and 20 days post-anthesis), using high-throughput RNA sequencing (RNA-Seq). We identified 427 grain-specific transcription factors (TFs) and 1653 differentially expressed TFs during grain development as well as a grain co-expression regulation network (GrainNet) of the TFs and their predicted co-expressed genes. Our study identified ten putative key TFs and the predicted regulatory genes of these TFs in wheat grain development of Xiaoyan-6. The analysis was given a firm basis through the study of additional wheat tissues, including root, stem, leaf, flag leaf, grain, spikes (from wheat plants at booting or heading stages) to provide a dataset of 92,478 high-confidence protein-coding genes that were mostly evenly distributed among subgenomes, but unevenly distributed across each of the chromosomes or each of the seven homeologous groups. Within this larger framework of the transcriptomes, we identified 4659 grain-specific genes (SEGs) and 26,500 differentially expressed genes (DEGs) throughout grain development stages tested. The SEGs identified mainly associate with regulation and signaling-related biological processes, while the DEGs mainly involve in cellular component organization or biogenesis and nutrient reservoir activity during grain development of Xiaoyan-6. This study establishes new targets for modifying genes related to grain development and yield, to fine-tune expression in different varieties and environments.

**Electronic supplementary material:**

The online version of this article (10.1007/s10142-019-00678-z) contains supplementary material, which is available to authorized users.

## Introduction

Allohexaploid common wheat (*Triticum aestivum* L., 2n = 6x = 42, AABBDD) has a complex genome with an overall size of 16 Gb (Appels et al. 2018; Zimin et al. 2017), and is one of the most important cereal crops for human diets worldwide. The increase in global population makes the improvement of wheat yield and quality a consistent and major target of wheat breeding. Wheat grain yield and quality is largely determined by the events occurring during wheat growth and development, and hence, the improvement of the traits of wheat yield and quality requires a better understanding of the biological processes in grain development and their regulation.

Wheat grain development can be broadly divided into three stages: cell division and expansion (0~14 days post-anthesis, DPA), effective grain filling (14~28 DPA), and maturation and desiccation (28 DPA to maturity) (Shewry et al. 2012). Final grain size and grain weight are largely determined by carpel size (Brinton et al. 2017; Calderini et al. 1999), which is established during the first two developmental stages when the basic structure of grain is generated and reserves (storage compounds like starch and gluten proteins) accumulation is nearly completed (Nadaud et al. 2010; Sabelli and Larkins 2009; Shewry et al. 2012). Grain development relies on gene expression regulated in a very strict chronological order.

Transcriptomics was used in our study to define events during the early grain development and grain filling stages to gain insights into the complex gene regulatory networks that underlies these specific phases. We used winter wheat cv. Xiaoyan-6, an elite Chinese cultivar that is the first cultivar successfully developed by crossing with *Agropyron elongatum* (2n=70) in the early stages of its pedigree, as research materials. Several studies have applied transcriptomics approaches to investigate the gene expression during grain development in wheat (Laudencia-Chingcuanco et al. 2007; Shewry et al. 2012; Wan et al. 2008; Yu et al. 2016). However, all these studies were conducted using microarrays, which represent a fraction of the transcriptome and are unable to distinguish between homoeologous genes. High-throughput RNA sequencing (RNA-Seq) has been extensively used to investigate grain development of cereal crops, such as *Oryza sativa* (Gao et al. 2013; Xue et al. 2012), *Zea mays* (Chen et al. 2014; Xiao et al. 2016), and wheat (Pfeifer et al. 2014). However, the accuracy of RNA-seq is dependent on the availability of a high-quality reference sequence and accurate gene models. All the studies mentioned above used either expressed sequence tags (ESTs) or the Chromosome Survey Sequence (CSS) (International Wheat Genome Sequencing Consortium 2014) as references. Recently, the releases of high-quality reference sequences and accurate annotations of hexaploid wheat (Appels et al. 2018; Clavijo et al. 2017; Zimin et al. 2017) have provided novel resources for the analysis of transcriptome from different tissues.

The winter wheat cultivar Xiaoyan-6 has high and stable yield as well as good quality for noodle and bread making, and it has been widely utilized as a donor parent in wheat breeding in China for approximately 30 years. However, the biological process and regulation of grain development of Xiaoyan-6 remain unclear. The main purpose of the present study is to reveal the changes in gene expression and to explore gene co-expression network of transcription factors (TFs) involved in wheat grain development of Xiaoyan-6. We investigated the global transcriptomes of Xiaoyan-6 spikes at two developmental stages and grains at four different developmental stages (5, 10, 15, and 20 DPA) using RNA-Seq, identified grain-specific genes (SEGs), and differentially expressed genes (DEGs) during developmental grains, and conducted GO annotation and KEGG enrichment of these genes. The analysis of vegetative tissues (root, stem, and leaf of five-leaf stage seedling, and flag leaf of wheat plant at heading stage) provided a useful reference point. We identified differentially expressed transcription factors (TFs) showing large changes in expression during grain development and established a grain co-expression regulatory network (GrainNet) by combining the high connectivity TFs with the genes that were predicted to be regulated by these TFs. The data in this study provides new insights into grain development of an elite donor parent in wheat breeding in China, based on defining functional genes associated with grain yield and quality of wheat.

## Materials and methods

### Plant materials and sample collection

Wheat cultivar Xiaoyan-6 was planted in the experimental station of Northwest A & F University, Yangling, Shaanxi, China (longitude 108° E, latitude 34° 15′ N), during a natural growth season in 2014 to 2015. Cultivation and management of wheat followed local normal production conditions. Ten tissues including root (R), stem (S), and leaf (L) of five-leaf-stage seedling (Zadoks 15) (Zadoks et al. 1974), flag leaf (FL) of wheat plant at booting stage (Zadoks 45), young spike (YS5) from wheat plant at booting stage (Zadoks 45), spike (YS15) from wheat plants at heading stages (Zadoks 53~54), and grains at 5, 10, 15, and 20 DPA (GR5, GR10, GR15, and GR20, respectively) were separately collected. Two independent biological replicates for each sample were collected for transcriptome sequencing, and three were collected for quantitative real-time reverse transcriptase PCR. The collected tissue samples were rapidly frozen in liquid nitrogen and then stored at − 80 °C until use.

### Total RNA isolation

Total RNA was extracted using Total RNA Rapid Extraction Kit for Polysaccharides Polyphenol Plant (BioTeke) according to the manufacturer’s directions. The quality and quantity of RNA samples were assessed by 1% RNase free agarose gel electrophoresis and NanoDrop 2000 Spectrophotometer (Thermo), respectively. The RNA samples passing the quality control were sent to Gene Denovo (Guangzhou, China) for cDNA library construction and sequencing on the Illumina sequencing platform (Illumina HiSeq™ 2500) after further assessment of accurate quantity of each RNA sample using Agilent 2100 Bio-analyzer (Agilent Technologies, Santa Clara, CA).

### cDNA library construction and transcriptome sequencing

cDNA libraries were constructed according to Illumina protocols. Briefly, mRNA was extracted using dynabeads oligo (dT) and fragmented by fragmentation buffer. Double-stranded cDNAs were synthesized using reverse-transcriptase and random hexamer primers. The cDNA fragments were purified using a QIA quick PCR extraction kit. These purified fragments were washed with EB buffer for end reparation of poly (A) addition and then ligated to sequencing adapters. Following agarose gel electrophoresis and extraction of cDNA from gels, the cDNA fragments were purified and enriched by PCR to construct the final cDNA library. The cDNA library was sequenced on the Illumina sequencing platform (Illumina HiSeq™ 2500) using the paired-end technology.

### Read alignment and expression quantification

Raw data of RNA sequencing were filtered using a Perl program to get the high-quality clean reads by removing low quality sequences (more than 50% bases with quality lower than 20 in one sequence), reads with more than 10% N bases (bases unknown), and reads containing adaptor sequences. The cleaned reads were aligned to two reference sequences from the same wheat accession (Chinese Spring) that were released at different time: the chromosome-based draft sequence of bread wheat (International Wheat Genome Sequencing Consortium 2014) and the IWGSC RefSeq v1.0 that is the latest released fully annotated reference genome of bread wheat (Appels et al. 2018). The tools used for read alignment and expression quantification included TopHat2 (TopHat 2.1.1, released on Feb. 23, 2016) (Kim et al. 2013) and Cufflinks (version 2.2.1, released on May 5, 2014) (Trapnell et al. 2010). To compare gene expression profiles across different tissues, the transcript levels of individual transcripts in each tissue were normalized as fragments per kilobase of transcript per million mapped reads (FPKM).

### Analysis of SEGs and DEGs in developmental grains

We defined a transcript/gene as an SEG if it is expressed only in grain rather than non-grain tissue. Identification of SEGs was conducted by comparing four types of tissue sample group, including RSL (the union of genes expressed in R, S, and L), FL, YS (the union of genes expressed in YS5 and YS15), and GR (the union of genes expressed in GR5, GR10, GR15, and GR20). Statistical tests were applied according to a method previously reported (Wang et al. 2015). DEGs in developmental grains were identified by comparing two consecutive time points (GR5 vs GR10, GR10 vs GR15, and GR15 vs GR20) using EdgeR (Robinson et al. 2010). The DEGs were required to show statistically significant low false discovery rate (FDR ≤ 0.001) and fold change of absolute value log_2_Ratio ≥ 1 (Wright and Simon 2003).

### Expression trend analysis of the DEGs during grain development

Gene expression trend analysis of DEGs during grain development was performed by Short Time-series Expression Miner software (STEM) (version 1.3.11, released on Dec. 26, 2016) (Ernst and Bar-Joseph 2006) on the OmicShare tools platform (www.omicshare.com/tools), a free online platform for data analysis. Before the trend analysis, relative expression level of individual genes across developmental grains was calculated as the logarithm (log_2_) of the fold change, whereas the fold change is the ration of the abundance of a gene in GR10, GR15, or GR20 to the abundance of the same gene in GR5. Then, the trend analysis was conducted. The clustered profiles with *P* value ≤ 0.05 were considered as significant profiles.

### Gene Ontology and Kyoto Encyclopedia of Genes and Genomes analysis

All the DEGs and the SEGs were subjected to Gene Ontology (GO) (http://geneontology.org/) to obtain GO annotation. GO annotation and enrichment analyses were conducted according to a protocol previously described (Zhang et al. 2013). GO enrichment analysis was conducted using the DEGs, the SEGs, and the genes from each expression trends, respectively, against the genes which were expressed across all tissues tested in Xiaoyan-6 (Benjamini and Hochberg 1995). Through the hypothesis test of the *P* value calculation, the GO terms with *P* value ≤ 0.05 were defined as significantly enriched GO terms. To visualize GO term enrichment, histograms were generated, where the top ten or 20 significantly enriched GO terms were displayed.

Kyoto Encyclopedia of Genes and Genomes (KEGG) (http://www.kegg.jp/) is a major public pathway-related database where functional classification and pathway assignment were provided. The DEGs and the SEGs were separately subjected to KEGG pathway enrichment analysis according to a method previously reported (Zhang et al. 2013). Pathways with *P* value ≤ 0.05 were defined as significantly enriched pathways.

### Weighted gene co-expression network analysis

Co-expression network analysis was performed using all the eight grain samples at four different developmental stages (GR5, GR10, GR15, and GR20), according to the protocol of the weighted gene co-expression network analysis (WGCNA) package in R (version 1.46, released on Mar. 28, 2015) (Langfelder and Horvath 2008), and then sample-clustering tree, modules, and kME (module eigengene-based connectivity) for individual genes were obtained. The parameters used in WGCNA were as follows: FPKM ≥ 1; cv (Variation of FPKM) ≥ 0.5; hierarchal clustering tree: dynamic hybrid tree cut algorithm; power: 27; minimum module size: 30; minimum height for merging modules: 0.15905. The genes with high degree of connectivity could be potentially major genes in the network. To explore major TFs and their co-expressed genes during wheat grain development, the TFs with high connectivity (kME ≥ 0.98) and their co-expression genes with top 100 edge weight were used to construct a co-expression network, then the network was visualized by Cytoscape (version 3.4.0, released on May 13, 2016). In the net, circular nodes represent genes and edges represent connection.

### Quantitative real-time reverse transcriptase-PCR (qRT-PCR)

Total RNA of individual tissues/organs was extracted using Total RNA Rapid Extraction Kit for Polysaccharides Polyphenol Plant (BioTeke) according to the manufacturer’s instructions. GoScript™ Reverse Transcription System (Promega) was used to synthesize cDNA. Three independent biological replicates were included. qRT-PCR was performed on CFX96 real-time PCR (Bio-Rad) with three replicates for each sample using GoTaq® qPCR Master Mix (Promega). Wheat *β-actin* (GenBank accession number: MF405765.1) was used as a reference gene, and the sequence of all the primers used in this study are listed in Supplementary Table 1. The values of the threshold cycle were analyzed according to the 2^−ΔΔCT^ method (Livak and Schmittgen 2001).

## Results and discussion

### The wheat cv. Xiaoyan-6 transcriptome

The wheat cv. Xiaoyan-6 transcriptome was characterized using RNA libraries from ten tissues sampled throughout the vegetative and reproductive stages, as well as the transition from vegetative to reproductive growth (Supplementary Table 2). Reads were aligned to the two reference sequences: the IWGSC CSS v2 and the IWGSC RefSeq v1.0, respectively. Consequently, 73% of reads were mapped to the CSS reference, while 85% of reads were mapped to the IWGSC RefSeq v1.0; as we expected, many more reads were mapped to the IWGSC RefSeq v1.0 (Supplementary Table 2). Consequently, 101,202 and 118,264 transcripts were identified when aligned to the CSS and the IWGSC RefSeq v1.0, corresponding to 84,520 and 92,478 annotated genes, respectively (Table 1). Considering the improved and more accurate gene annotations provided by IWGSC Refseq v1.0 rather than the CSS gene models, we continued our data analysis only using the results of the alignment to the IWGSC RefSeq v1.0. Consequently, a total of 92,478 protein-coding genes were detected in the ten tissues tested (in duplicate), covering 83.47% (92,478/110,790) of the annotated high-confidence protein-coding genes in the reference (Table 1).

**Table 1:** Summary of wheat cv. Xiaoyan-6 RNA Sequencing data from ten tissues analyzed using two different gene models of wheat cv. Chinese Spring as references

Data source	Raw reads data^*4^	Clean reads data^*4^	Gene number	Transcript number
Wheat cv. Xiaoyan-6^*1^	184.37 Gb	180.59 Gb	92,478^*5^	118,264^*5^
			84,520^*6^	101,202^*6^
IWGSC RefSeq v1.0^*2^	–	–	110,790	137,056
IWGSC CSS v2^*3^	–	–	100,934	112,496

^*1^A Chinese winter wheat cv. Xiaoyan-6 used in the present study

^*2^IWGSC RefSeq v1.0, the fully annotated reference genome sequence of hexaploid wheat cv. Chinese Spring (Appels et al. 2018)

^*3^IWGSC CSS v2 presents genome sequence of wheat cv. Chinese Spring: the Chromosome Survey Sequence reference (International Wheat Genome Sequencing Consortium 2014)

^*4^1 Gb is equal to ten to the ninth power bp (1 Gb = 10^^9^ bp)

^*5^Gene or transcript number using the IWGSC RefSeq annotation v1.0 as a reference

^*6^Gene or transcript number when using the IWGSC CSS v2 as a reference

The heatmap of sample correlations, which based on the expression values (FPKM) of all genes in the 20 samples tested (Supplementary Table 3), showed good reproducibility between the two biological replicates (Supplementary Fig. 1a and Supplementary Fig. 1b). Principal component analysis (PCA) showed that the first principal component could explain 49.6% of total variance and distinguish samples based on tissues identity, discriminating developmental grains (GR5, GR10, GR15, and GR20) from spikes (YS5 and YS15) with vegetative tissues R, S, L, and FL in between; and the second principal component could explain 24.5% of the total variance and separate organs according to developmental stages (Supplementary Fig. 1c). Cluster analysis classified tissue samples into three groups corresponding to their developmental stages, suggesting similar expression patterns in similar tissues (Supplementary Fig. 1c).

To further validate the RNA-Seq data, 20 genes randomly selected were quantified for their expression profiles across all the tested tissues through qRT-PCR with homoeolog-specific primers, including three SEGs (TraesCS2B01G392500, TraesCS2A01G013400, and TraesCS4B01G013700) and 17 constitutively (all tissues) expressed genes, *NAC* (TraesCS2A01G338300), *MYB* (TraesCS3A01G336500), and *MIKC_MADS* (TraesCS5B01G286100) also being included (Supplementary Fig. 2 and Supplementary Table 3). It can be shown that the expression patterns of these 20 genes quantified by qRT-PCR were highly consistent with those obtained by RNA-Seq (Supplementary Fig. 2), demonstrating that the RNA-Seq data are reliable.

**Fig. 1:**
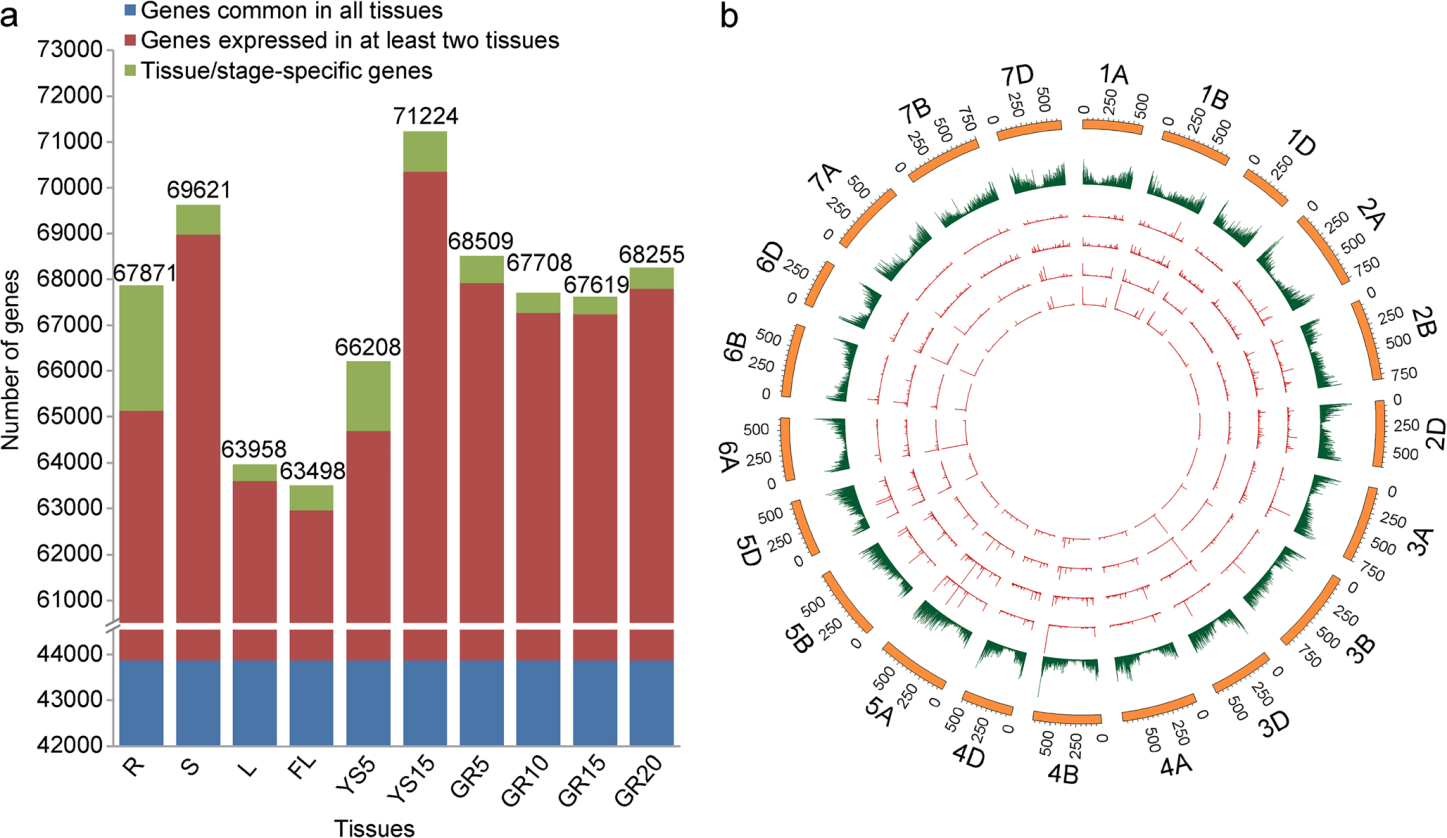
Overview of the transcriptomes of wheat cv. Xiaoyan-6. **a** Number of genes detected in each of the ten tissues tested. The numbers above individual columns present total number of genes detected in the corresponding tissues. The detail number of the genes common in all tissues, the genes expressed in at least two tissues, and tissue or developmental stage-specific genes were shown in Supplementary Table 4. R, S, and L represent root, stem, and leaf of five-leaf stage seedling, respectively. FL represents flag leaf of wheat at heading stage. YS5 represents young spike of wheat plant at early booting stage. YS15 represents spike of wheat plant at heading stage. GR5, GR10, GR15, and GR20 represent grain at 5, 10, 15, and 20 days post-anthesis, respectively. **b** Circular diagrams of the distribution and the genomic characteristics of all the expressed genes across 21 wheat chromosomes. From outside to inside, each circle represents chromosome name and size (50-Mb tick size), gene density (0 to 47 gene per Mb), and gene expression values (0 to 21,163 FPKM) in grain samples (GR5, GR10, GR15, and GR20, respectively). The length of the line in circles represents the value, FPKM means fragments per kilobase of transcript per million mapped reads

The overview of different wheat tissue/organ transcriptomes is shown in Fig. 1a and Supplementary Table 4. The average of 67,447 genes was detected in the ten tissues tested, with the least number in FL (63,498) and the most in YS15 (71,224) (Fig. 1a and Supplementary Table 4). About 47.4% (43,871/92,478) of the detected genes were common in all these tissues (Supplementary Table 4), and GO categories of these common genes were over-represented for cellular nitrogen compound metabolic process, cellular biosynthetic process, gene expression, cellular localization, intracellular transport, establishment of localization in cell, protein localization, or organelle organization (Supplementary Table 5), suggesting that these genes might play general functions essential for the cells of all these tissues. In contrast, only 0.38~2.96% (354/92,478~2734/92,478) of the total genes were tissue-specific or development stage-specific (Fig. 1a and Supplementary Table 4). Moreover, among the vegetative tissue, root had the greatest number of tissue-specific genes (2734), which is approximately 2 to 7 times greater than those of the other tissues (Fig. 1a and Supplementary Table 4). This probably reflects large differences in gene expression profiles between underground and aerial organs. For example, wheat germ agglutinin genes are root-specific, which play an important role in the colonization of plant growth promoting rhizobacteria, enhancing plant growth, preventing disease, and increasing crop yield (Kloepper et al. 1980; Rodriguez-Navarro et al. 2007).

To characterize the subgenome and chromosome distribution of all the expressed genes across wheat tissues, the data of individual gene location and gene expression value in the grain samples (GR5, GR10, GR15, and GR20) were compared across each of the 21 chromosomes (Fig. 1b and Supplementary Table 6), with the 1803 genes of unknown origin (with gene/transcript ID of “TraesCSU” in Supplementary Table 3) being excluded. These expressed genes covered 82.78% (30,050/36,302), 83.19% (30,563/36,738), and 85.84% (30,062/35,021) of the total reference genes in A, B, or D subgenome of the IWGSC RefSeq v1.0, respectively (Supplementary Table 6). Furthermore, the expression levels of genes across individual chromosomes were similar across the grain developmental stages of Xiaoyan-6 (the four most inner circles in Fig. 1b), with exceptions of homoeologous chromosome groups I (1A/1B/1D), II (2A/2B), and VI (6A/6B/6D) that had at least one hotspot (with a median expression level across the grain developmental stages more than 30 FPKM, containing at least five genes) (Supplementary Table 6). It was previously reported that the expression levels of genes across chromosomes of wheat cv. Chinese Spring (CS) were also similar, with the exception of 19 genetic bins that had “hotspots” (with a median expression level > 20 tpm, containing on average 5 genes) across the six tissues, including leaf, root, seedling, stem, spike, and seed (Clavijo et al. 2017). The difference of gene expression distribution across subgenomes and chromosomes between Xiaoyan-6 and CS most likely reflects the differences in the genomes between Xiaoyan-6 and CS (Li et al. 1990). It was demonstrated that Xiaoyan-6 lineage involved an *A. elongatum* cross at the early stages of its pedigree, which resulted in the presence of at least two *Agropyron* chromosome segments within five chromosome arms of Xiaoyan-6, 1AL, 2AS, 5AS, 6AS, and 7BS. In addition, at least two reciprocal interchanges involved in 1A, 2D, 3B, 4D, and 6A present in Xiaoyan-6 (Li et al. 1990).

The high-quality and fully annotated wheat genome sequence IWGSC Refseq v1.0 provided an opportunity to analyze homoeolog-specific gene expression. Transcript levels of all triads were analyzed across the ten tissues tested. It was found that almost one-third of expressed triads exhibited unevenly unbalanced expression among three subgenomes across the tissues of Xiaoyan-6, taking *TaCYP78A* family members (*TaCYP78A3, A5, A12, and A16*) as examples (Supplementary Fig. 3a–d). The homoeologous triads of *TaCYP78A3* (TraesCS7A01G270700, TraesCS7B01G168800, and TraesCS7D01G271100) showed higher expression level in D and B subgenome across tissues (Supplementary Fig. 3a and Supplementary Table 3); the triads of *TaCYP78A5* (TraesCS2A01G175700, TraesCS2B01G201900, and TraesCS2D01G183000) showed unevenly unbalanced expression pattern among three subgenomes across tissues and developmental stages (Supplementary Fig. 3b and Supplementary Table 3); the triads of *TaCYP78A12* (TraesCS5A01G316600, TraesCS5B01G317200, and TraesCS5D01G322900) exhibited higher expression level in A and D subgenome (Supplementary Fig. 3c and Supplementary Table 3), while the expression level of three triads of *TaCYP78A16* (TraesCS5A01G502000, TraesCS4B01G330500, and TraesCS4D01G327400) were relatively balanced among subgenomes across the tissues (Supplementary Fig. 3d and Supplementary Table 3). Similar unbalanced expressions were also observed in Chinese Spring (Clavijo et al. 2017) and Azhurnaya (Ramirez-Gonzalez et al. 2018).

In general, a total of 92,478 protein-coding genes were expressed across the ten tissues tested (in duplicate) of wheat cv. Xiaoyan-6; almost half of the total expressed genes were common in all the tissues tested, which suggested that these genes play general functions essential for cells, while ~ 2.96% were tissue-specific or development stage-specific. In addition, the expressed genes across these tissues covered 82.78%, 83.19%, and 85.84% of the total reference genes in A, B, or D subgenome of the IWGSC RefSeq v1.0, respectively, and unevenly distributed within each of the seven homoeologous chromosome groups. Expression levels of genes across individual chromosomes were similar across the grain developmental stages of Xiaoyan-6, with the exception of homoeologous chromosome groups I (1A/1B/1D), II (2A/2B), and VI (6A/6B/6D) that had at least one hotspot (with a median expression level across the grain developmental stages more than 30 FPKM, containing at least five genes). These data complement previous findings on tissue-specific gene expression and gene distribution patterns among subgenomes and chromosomes in hexaploid wheat.

### Expression trends and GO enrichment of the genes involved in grain development

**Fig. 2:**
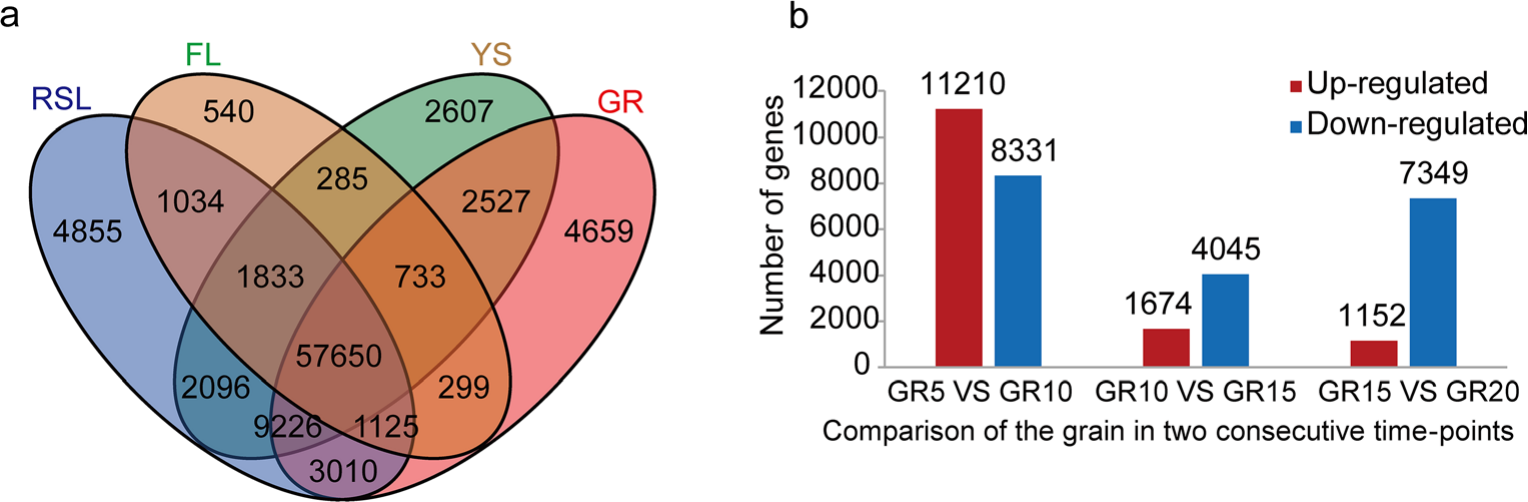
Distribution of the expressed genes in different types of wheat tissues/organs and differentially expressed genes in developmental grains. **a** Venn diagram showing the distribution of the expressed genes in four types of sample group, including RSL, FL, YS, and GR. RSL, vegetative tissues from seedling including root, stem, and leaf of wheat plant at five-leaf stage; FL, flag leaf of wheat at heading stage; YS, spike from wheat plant at booting stage and heading stage; GR, grains at 5, 10, 15, and 20 days post-anthesis. **b** Histogram of differentially expressed genes in developmental grains by comparing two consecutive time points. The criteria for differential expression: false discovery rate (FDR ≤ 0.001) and fold changes (absolute value of log_2_Ratio ≥ 1)

Among 92,478 protein-coding genes detected in the ten wheat tissues tested, 79,229 (86%) were identified as being expressed in developmental grains by unifying all the genes expressed in the four grain samples (GR5, GR10, GR15, and GR20) (Supplementary Table 4, Fig. 2a). Further, 4659 SEGs were identified by comparing four types of tissue sample group as described in “Materials and methods” (Fig. 2a), and the results of statistical tests illustrated the significance of the intersections (Supplementary Fig. 4). DEGs were also identified by comparing two consecutive time points of grain development stages, and the results indicated that large numbers of genes (26,500) exhibited differential expression throughout the grain development stages tested and the majority of these variations take place during the early stage of grain development (between 5 and 10 DPA) (Fig. 2b), this is corresponding to the early developmental and metabolic events occurring in the grain of wheat (Shewry et al. 2012) as well as being consistent with the results obtained by using cDNA arrays (Laudencia-Chingcuanco et al. 2007). However, the numbers of the DEGs between two consecutive time points in the present study were much greater than those in previous reports, most likely owing to the improved transcriptome reference and sensitivity of the RNA-Seq methodology. GO and KEGG annotations as well as the pathway mapping of all the SEGs and the DEGs were summarized in Table 2, Supplementary Table 7, Supplementary Table 8, Supplementary Fig. 5, and Supplementary Fig. 6. These data indicated that GO terms were over-represented for the SEGs encoding the components of regulation and signaling-related categories, and GO categories were over-represented for the DEGs encoding the components of cellular component organization or biogenesis and nutrient reservoir activity during grain development of Xiaoyan-6. In contrast, the GO terms of the genes common in all the tissues tested were over-represented for those genes encoding the proteins involved in general cellular biological process (Supplementary Table 5). A recent study in wheat cv. Chinese Spring indicated that “hotspots” (with a median expression level > 20 tpm, containing on average 5 genes) across the six tissues, including leaf, root, seedling, stem, spike, and seed, tended to be enriched for genes encoding components of cellular component organization or biogenesis in all the tissues as well as nutrient reservoir activity in seed tissues (Clavijo et al. 2017).

**Table 2:** Summary of GO enrichment of the grain-specific genes (SEGs) and the differentially expression genes (DEGs) as well as their pathways during wheat grain development grouped based on metabolic activities

GO/KEGG analysis	DEGs	SEGs
GO enrichment		
Cellular component	318	10
Molecular function	504	68
Biological process	1285	122
Metabolic activity		
Amino acid metabolism	6	2
Biosynthesis of other secondary metabolites	1	3
Carbohydrate metabolism	3	3
Energy metabolism	2	0
Environmental adaptation	0	2
Folding, sorting and degradation	1	2
Glycan biosynthesis and metabolism	1	1
Lipid metabolism	4	1
Metabolism of cofactors and vitamins	1	0
Signal transduction	0	1
Replication and repair	2	0
Translation	3	0

For the list of enriched GO categories and various metabolic pathways that are grouped under various metabolic activities, please refer to Supplementary Table 7 and Supplementary Table 8, respectively

**Fig. 3:**
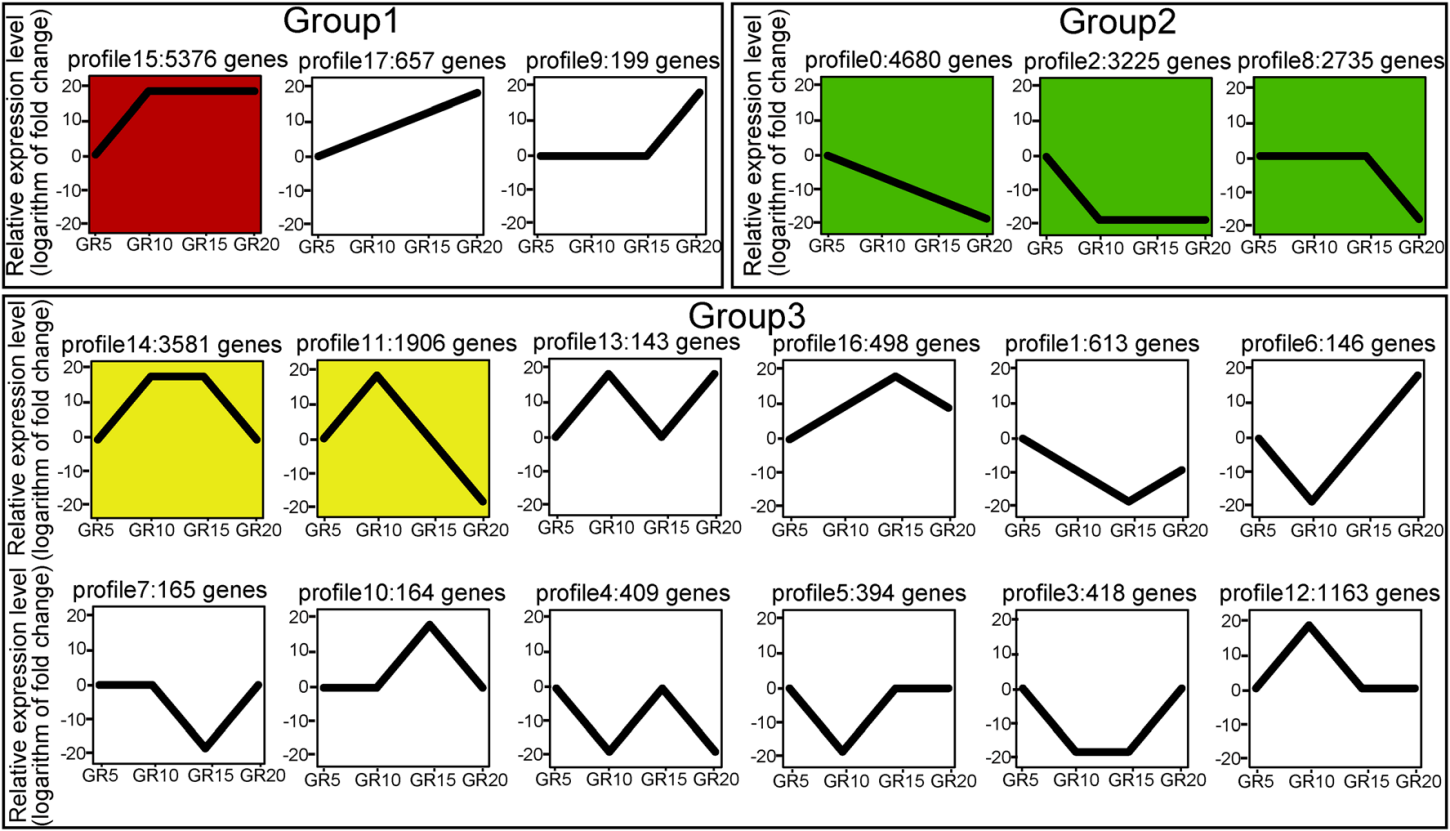
Expression trends of differentially expressed genes during wheat grain development (DEGs). Each panel represents a model of an expression file. The vertical axes represent relative expression levels (the logarithm (log_2_) of the fold change, whereas the fold change is the ration of the abundance of a gene in GR10, GR15, or GR20 to the abundance of the same gene in GR5) across the developmental stages of wheat grain. The horizontal axes indicate developmental grains; GR5, GR10, GR15, and GR20 representing grain at 5, 10, 15, and 20 days post-anthesis, respectively. The DEGs are clustered to 18 expression profiles according to shared expression patterns, which are classified into three groups: groups 1, 2, and 3 corresponding to the trends of up-regulated, down-regulated, and modulated (up-regulation to down-regulation or vice versus) with grain development, respectively. The gene number containing in each expression profile is listed following the profile number. Six statistically significant expression profiles (with *P* ≤ 0.05) are marked with colors

The DEGs were grouped according to shared expression patterns during grain development (Supplementary Table 7). Expression trend analysis of these DEGs exhibited 18 expression profiles being classified into three groups: up-regulated (group 1), down-regulated (group 2), and modulated with grain development (group 3) (Fig. 3, Supplementary Fig. 7, and Supplementary Table 7), providing the context for understanding the complex metabolic pathways and the molecular control of the quality and nutrition properties of Xiaoyan-6. Among the 18 expression profiles, six were statistically significant (*P* ≤ 0.05) expression trends (profiles 15, 0, 2, 8, 14, and 11), and each of these significant expression trends tended to be associated with those biological processes and molecular functions as shown in Supplementary Fig. 8. The profile 15 belongs to the up-regulated group, and the genes in this profile are increasingly expressed with grain development and remain at a stable level after 10 DPA (Fig. 3, Supplementary Fig. 7). The most enriched GO terms in this profile are related to regulation of biological or metabolic process (Supplementary Fig. 8). This is in line with the metabolic processes that are very active at the early developmental stages of wheat grain. Profiles 0, 2, and 8 belong to the down-regulated group, and the most enriched GO terms in these profiles are associated with photosynthesis and secondary metabolic processes (Supplementary Fig. 8). This is consistent with the cellular events taking place during growth and development of wheat grain.

Taken together, the above findings in Xiaoyan-6 that the GO terms of the SEGs were over-represented in regulation and signaling-related biological processes and that the individual expression trends of the DEGs during grain development tended to be associated with certain biological processes provide new information to the existing research on wheat transcriptome and enrich our knowledge of functional genes involved in wheat grain development.

### TFs involved in wheat grain development

TFs regulate target genes to ensure tightly regulated developmental process. A total of 3606 wheat TFs in 56 families have been collected in Plant Transcription Factor Database (PlantTFDB 4.0, http://planttfdb.cbi.pku.edu.cn/index.php) (Jin et al. 2017). In the present study, we detected 4735 TFs expressed in grain samples from all 56 families that were identified using the Transcription Factor Prediction tool in PlantTFDB through Hidden Markov Model-guided method (Jin et al. 2017). We also found 427 grain-specific TFs (called as SE-TFs) and 1635 TFs differentially expressed during grain development (named as DE-TFs) (Table 3 and Supplementary Table 9). Among the 1635 DE-TFs, 425 and 749 showed expression patterns of group 1 and group 2, respectively (Table 3 and Supplementary Table 9), corresponding to the up-regulated and the down-regulated groups as shown in Fig. 3. By comparison, relatively few of TFs differentially expressed through wheat grain development were identified previously by cDNA microarrays technique (Laudencia-Chingcuanco et al. 2007; Wan et al. 2008), and the previously identified TFs were represented in our set of TFs as summarized in Supplementary Table 10. In rice, 1118 TFs from 55 families were also detected in developmental endosperm using RNA-Seq technique (Gao et al. 2013). A comparison of the TF families previously identified in rice development endosperm with our set of developmental grain TF families (Supplementary Table 10) detected similar TF families (56). One-third of the wheat grain TF genes had close homologs (70% identity, 70% coverage) in rice (Supplementary Table 10).

**Table 3:** Statistics of transcription factors (TFs) and their expression patterns

TF family^*1^	The number of TFs across tissues	The number of TFs in grain	SE-TFs^*2^	DE-TFs^*3^	DE-TFs in three expression pattern^*4^		
					Group 1	Group 2	Group 3
AP2	71	68	5	19	8	3	8
ARF	66	66	0	30	8	12	10
ARR-B	46	46	23	11	2	1	8
B3	280	237	40	60	24	27	9
BBR-BPC	6	6	0	1	1	0	0
BES1	20	18	1	9	2	6	1
bHLH	476	365	9	108	22	65	21
bZIP	266	243	10	85	42	21	22
C2H2	354	312	34	96	18	31	47
C3H	126	124	5	47	12	6	29
CAMTA	16	16	0	2	0	1	1
CO-like	44	37	1	15	0	9	6
CPP	35	32	2	9	5	0	4
DBB	26	26	0	13	3	5	5
Dof	95	91	3	36	12	19	5
E2F/DP	27	27	0	11	2	3	6
EIL	19	19	1	8	5	3	0
ERF	437	367	46	157	15	92	50
FAR1	137	128	40	19	17	0	2
G2-like	144	125	5	32	11	15	6
GATA	77	73	5	16	2	4	10
GeBP	31	31	0	10	0	2	8
GRAS	156	135	12	44	6	29	9
GRF	29	29	0	13	2	4	7
HB-other	52	50	23	23	19	2	2
HB-PHD	9	9	0	5	4	0	1
HD-ZIP	120	104	6	51	3	39	9
HRT-like	2	2	0	0	0	0	0
HSF	77	73	2	27	7	9	11
LBD	79	62	5	24	1	16	7
LFY	3	2	0	0	0	0	0
LSD	13	13	0	4	0	4	0
MIKC_MADS	106	101	5	34	0	27	7
M-type_MADS	95	76	27	2	0	2	0
MYB	410	352	19	136	37	63	36
MYB_related	216	174	32	70	42	8	20
NAC	429	342	36	125	36	63	26
NF-X1	7	7	0	3	2	0	1
NF-YA	19	19	0	10	9	0	1
NF-YB	53	47	11	25	11	1	13
NF-YC	39	38	2	11	3	6	2
Nin-like	32	30	1	6	3	0	3
RAV	26	23	0	5	0	4	1
S1Fa-like	3	3	0	3	0	0	3
SBP	56	41	0	11	3	7	1
SRS	15	14	0	5	1	3	1
STAT	3	3	0	1	1	0	0
TALE	65	62	0	17	0	9	8
TCP	59	48	0	18	0	16	2
Trihelix	91	90	1	40	8	22	10
VOZ	6	6	0	3	2	0	1
Whirly	6	6	0	2	0	0	2
WOX	43	32	9	9	4	1	4
WRKY	287	234	4	107	6	75	26
YABBY	20	19	0	10	0	9	1
ZF-HD	35	32	2	15	4	5	6
Total	5460	4735	427	1653	425	749	479

^*1^TFs, transcription factors

^*2^SE-TFs, TFs specially expressed in developmental grains

^*3^DE-TFs, TFs differentially expressed during grain development

^*4^Group 1, group 2, and group 3 are corresponding to the groups shown in Fig. 3, which represent up-regulated, down-regulated and modulated with grain development, respectively

### Gene co-expression network and key TFs involved in grain development

WGCNA is one of the most widely used approaches aimed at the systematic understanding of network instead of individual genes (Langfelder and Horvath 2008). To explore the gene co-expression regulation network and determine the putative key genes in the regulatory pathway, we applied WGCNA across all eight grain samples from the four developmental stages, and a hierarchical clustering tree with 15 distinct transcription modules was constructed after merging of similar modules (Supplementary Fig. 9). The correlation between modules and different grain developmental stages showed that module 3 and module 4 are positive correlated with GR5 and GR10; module 6 and module 11 are positive correlated with GR15 or G20; while module 5 and module 13 are negative correlated with GR15 or G20 (Supplementary Fig. 10). The connectivity values of individual genes and their expression pattern modules are presented in Supplementary Table 11.

**Fig. 4:**
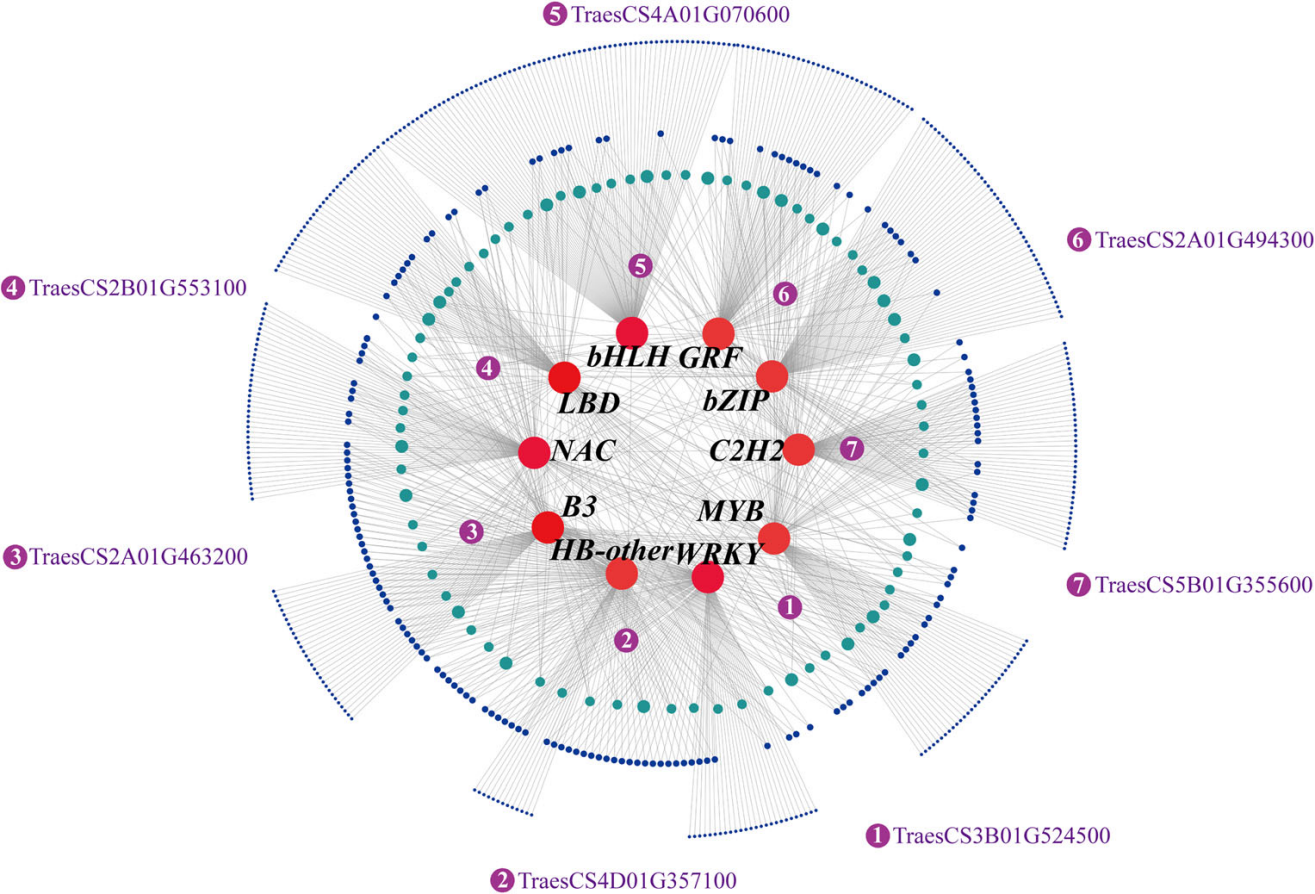
Wheat grain gene co-expression network (GrainNet). The GrainNet was developed by using the transcription factors (TFs) genes with high module eigengene-based connectivity (kME ≥ 0.98) and their putative regulatory genes with top 100 edge weight. Circular nodes represent genes in the net, the size of nodes represents the connectivity, and the edges represent interaction between TF genes and their predicted regulatory genes. The nodes with more edges indicate more importance in the network. The group of the red nodes represent TF genes, the group of the purple nodes represent the genes predicted to be regulated by five or six of these TFs and the gene IDs are shown outside the circles, the group of the light blue ones represent the genes predicted to be regulated by three or four of these TFs, and the group of the dark blue nodes represent the genes predicted to be regulated by one or two of these TFs

As described above, 4735 of the total 5460 TFs identified across the tissues tested were expressed in developmental grains of wheat cv. Xiaoyan-6 (Table 3). We developed a grain co-expression regulation network (GrainNet) by using the TF genes with high connectivity and their putative regulatory genes with high edge weight in order to identify the putative critical TFs and functional genes involved in wheat grain development (Fig. 4 and Supplementary Table 12). In the GrainNet, ten TF genes (red nodes in Fig. 4) from different families, including *bZIP* (TraesCS2B01G489900), *C2H2* (TraesCS4A01G041400), *MYB* (TraesCS3B01G399300), *WRKY* (TraesCS2A01G489500), *HB-other* (TraesCS7A01G168000), *B3* (TraesCS4A01G055700), *NAC* (TraesCS5D01G148800), *LBD* (TraesCS2A01G271300), *bHLH* (TraesCS1D01G084200), and *GRF* (TraesCS4A01G255000), are considered to be the top ten highly connected TFs (hub TFs) in the GrainNet (Fig. 4 and Supplementary Table 11). The other nodes represent the predicted regulatory genes of these key TFs, the purple nodes indicating the genes predicted to be regulated by five or six of these TFs, the light blue ones indicating the genes predicted to be regulated by three or four of these TFs, and the dark blue ones representing the genes predicted to be regulated by one or two of the TFs. In general, TFs and their predicted regulatory genes showed a many-to-many relationship, multiple TFs regulating any one predicted gene and individual TFs regulating multiple predicted genes.

To explore the major biological functions of the key TFs involved in grain development, we first analyzed expression profiles of the ten putative key TFs and the seven predicted target genes of these TFs (the purple nodes in Fig. 4) across all wheat tissues tested. The result exhibited that nine of the ten TFs and four of the seven functional genes most highly expressed during early grain development (Supplementary Fig. 11), suggesting that they play important roles in early grain development of Xiaoyan-6. Then, we focused on the molecular functions of the seven predicted target genes of these TFs. TraesCS3B01G524500 encodes expansin B7, a β-expansin that affects leaf elongation, internodal elongation, and grain development by regulating the extension of cell wall in rice (Lee and Kende 2001; Xu et al. 2016), and it is highly expressed during very early grain development of Xiaoyan-6 (Supplementary Fig. 11), these suggested that this gene might be play an important role in cell expansion in early developmental wheat grain. TraesCS4D01G357100 encodes nucleoside triphosphatase (NTPase), an important membrane enzyme participating in substance trans-membrane transport, elongation growth of cells as well as responses to environmental stress (Dietrich et al. 2001; Rober-Kleber et al. 2003; Serrano 1988). TraesCS4D01G357100 is most highly expressed in early grain development (Supplementary Fig. 11). TraesCS2A01G463200 encodes BCL2-associated athanogene (*BAG*) family molecular chaperone regulator 1-like that can interact with molecular chaperones directly and play a role in a variety of signaling pathways (Brive et al. 2001). *BAG1* in rice is involved in cell elongation and cell cycle regulation (Kabbage and Dickman 2008), indicating that TraesCS2A01G463200 could also be involved in signaling, cell division, expansion, differentiation, and elongation of endosperm cells during the early grain development stage and grain filling of wheat. TraesCS2B01G553100 encoding alpha-L-arabinofuranosidase/beta-D-xylosidase isoenzyme ARA-I was demonstrated to affect nutritional quality and processing quality of wheat grain by controlling the content of non-starch polysaccharide in grain cell wall (Cleemput et al. 1997). TraesCS4A01G070600 encoding UDP-glycosyltransferase 83A1 (*UGT83A1*) can catalyze the glycosylation and play an important role in the modification of secondary metabolites (Zhang et al. 2014). TraesCS2A01G494300 can produce small auxin up RNA 19 (*SAUR19*). *SAUR* is a plant-specific protein family and also the largest family in auxin-responsive factors. *AtSAUR19* in Arabidopsis is capable to positively modulate cell expansion by regulating auxin synthesis and transport (Spartz et al. 2012). While, TraesCS5B01G355600 encoding pentatricopeptide repeat-containing protein (PPR-like) in mitochondrial is expressed at a low level during grain development (Supplementary Fig. 11). The homolog of this PPR-like gene in Arabidopsis is At5g41170, and its loss-of-function mutant plants result in some abnormal phenotypes, such as development retardation, flowering delay, plant smaller, leaf curl, mature seed volume reduction, seed coat shrinkage, embryo or endosperm deficiency (Liu et al. 2013; Manavski et al. 2012). These infer that TraesCS5B01G355600 might play an important role during wheat plant development. Moreover, we also performed GO enrichment analysis on the genes predicted to be regulated by two or more of the putative key TFs, and the result showed that the GO terms of signal transduction, amino acid transport and metabolic process, cell growth, and hormone transport were over-represented (Supplementary Table 13). Taken together, these predicted functional genes now define network regulated by multiple putative key TFs associated with signaling, cell cycle regulation, cell elongation and expansion, metabolite trans-membrane transport, and the components of the cell wall, this implying their crucial roles in early grain development of wheat cv. Xiaoyan-6.

A previous study in wheat cv. Chinese Spring developed a co-expression network based on homeologous gene expression in different cell types (starchy endosperm, aleurone layer, transfer cells) at different developmental stages of grain (10, 20, 30 DPA) and revealed that the co-expression network consisted of 25 modules displaying distinct co-expression clusters associated with the spatiotemporal progression during endosperm development (Pfeifer et al. 2014). In our study, the co-expression network consisting of TFs and their predicted regulatory genes was developed based on gene expression at different development stages of wheat grain (5, 10, 15, and 20 DPA), using the latest released fully annotated reference genome of bread wheat (Appels et al. 2018) as a reference. The GrainNet in the present study focused on the TFs and their predicted regulatory genes involved in developmental grains of wheat and allowed identification of the putative critical TFs and functional genes, compared to the previous work. Our GrainNet provides new insights into gene co-expression regulation network during grain development of wheat. Our findings establish new target genes for further study of the functional genes related to grain development and yield and for modifying genes related to grain development and yield, to fine-tune expression in different varieties.

## Conclusion

In this study, we investigated the transcriptome of Chinese winter wheat cv. Xiaoyan-6 grains at four developmental stages (5, 10, 15, and 20 DPA) using RNA-Seq, with the IWGSC RefSeq ver1.0 as a reference, to identify 427 SE-TFs and 1653 DE-TFs as well as a GrainNet consisting of the TFs and their predicted regulatory genes that are first being developed in wheat. Previously, in wheat cv. Chinese Spring, a co-expression network consisting of 25 modules that displayed distinct co-expression clusters associated with the spatiotemporal progression during endosperm development was developed based on homeologous gene expressions in different cell types (starchy endosperm, aleurone layer, transfer cells) at three different developmental stages of grain (10, 20, and 30 DPA), using the IWGSC CSS v2 as a reference. In the present study, the GrainNet based on co-expression network focused on the TFs and their predicted regulatory genes involved in developmental grains of wheat and allowed to identify ten putative key TFs and the predicted regulatory genes of these TFs in developmental grain of Xiaoyan-6, compared to the work in Chinese Spring. The analysis was given a firm basis through the study of additional wheat tissues, including root, stem, leaf, flag leaf, and spike at two developmental stages to generate a dataset of 92,478 high-confidence protein-coding genes that were mostly evenly distributed among subgenomes but unevenly distributed across each of the seven homoeologous chromosome groups. Moreover, the expression levels of the genes across chromosomes are similar across developmental grains of Xiaoyan-6, with the exception of homoeologous chromosome group I (1A/1B/1D), II (2A/2B), and VI (6A/6B/6D) that had at least one hotspot (with a median expression level across developmental grains more than 30 FPKM, containing at least five genes). Within this larger framework, the transcriptomes identified 4659 SEGs and 26,500 DEGs throughout grain development stages tested. The SEGs were mainly associated with regulation and signaling-related biological processes, and the DEGs were mainly involved in cellular component organization or biogenesis and nutrient reservoir activity during grain development of Xiaoyan-6. Gene expression trend analysis of the DEGs revealed six statistically significant expression profiles during grain development of wheat. The study establishes new targets for further study of functional genes related to grain development and yield and for modifying genes related to grain development and yield, to fine-tune expression in different varieties. All the raw data in our study has been deposited in NCBI’s Sequence Read Archive under BioProject number PRJNA525250.

## Electronic supplementary material

Supplementary Fig. 1 Analysis of the relationship among all 20 wheat samples according to RNA-seq based gene expression values. R, S, and L represent root, stem and leaf of five-leaf stage seedling, respectively. FL represents flag leaf of wheat plant at heading stage. YS5 represents young spike of wheat at early booting stage. YS15 represents spike of wheat at heading stage. GR5, GR10, GR15, and GR20 represent grain at 5, 10, 15, and 20 days post-anthesis, respectively. **a** Expression density plots of all samples. FPKM, fragments per kilobase of transcript per million mapped reads. **b** The heatmap of sample correlation. The bar in right represents the scale of the relationship among samples, and the value in each pane represents the correlation coefficient between two samples. **c** Principal components analysis (PCA). All samples were clustered into three separate groups corresponding to vegetative tissues, developing spikes, and developing grains shown by green, red and gray colors, respectively. The numbers in parentheses represent the proportion of variance explained by that principal component. (PNG 455 kb)

High resolution image (TIF 1693 kb)

Supplementary Fig. 2 Comparison of the gene expression patterns determined by quantitative real-time RT-PCR (qRT-PCR) and RNA-seq. The horizontal axis is ten wheat tissues/organs. R, S, and L represent root, stem, and leaf of five-leaf stage seedlings, respectively, FL represents flag leaf of wheat plants at heading stage, YS5 represents young spike of wheat plant at early booting stage, YS15 represents spike of wheat plant at heading stage, GR5, GR10, GR15, and GR20 represent grain at 5, 10, 15, and 20 days post-anthesis, respectively. The left vertical axes show the relative expression levels of the tested gene in individual tissues obtained by qRT-PCR and correspond to histogram, the maximum gene expression levels being defined as one. The right vertical axes are FPKM of the tested gene in individual tissues resulted from RNA-seq and correspond to line chart, whereas FPKM means fragments per kilobase of transcript per million mapped reads. The error bars on the histogram represent the standard deviation of three biological replicates, and the error bars on the line chart represent the standard deviation of two biological replicates. The annotations of these genes were listed in Supplementary Table 3. (PNG 1148 kb)

High resolution image (TIF 3805 kb)

Supplementary Fig. 3 Expression patterns of the homoeologous triads of *TaCYP78A* family members across wheat tissues/organs based on RNA-seq data. R, S, and L represent root, stem, and leaf tissue of five-leaf stage seedling, respectively. FL represents flag leaf of wheat plant at heading stage. YS5 represents young spike of wheat at early booting stage. YS15 represents spike of wheat at heading stage. GR5, GR10, GR15, and GR20 represent grain at 5, 10, 15, and 20 days post-anthesis, respectively. The number 1 or 2 the behind the names of tissue sample indicates two biological repeats, respectively. The Y axes show the relative expression levels of the tested gene in individual tissues. The numbers within brackets are the corresponding gene ID from the IWGSC RefSeq v1.0, the latest released fully annotated reference genome of bread wheat, their expression values and annotations being shown in Supplementary Table 3. (PNG 142 kb)

High resolution image (TIF 415 kb)

Supplementary Fig. 4 Visualization of the intersections among four types of tissue sample group RSL, FL, YS, and GR. A circular plot illustrate all possible intersections and the corresponding statistics. RSL indicates the union of the genes expressed in root, stem, and leaf tissue, FL represents flag leaf of wheat plants at heading stage, YS represents the union of the genes expressed in young spike of wheat plants at early booting stage and at heading stage, GR indicates the union of the genes expressed in grains at 5, 10, 15, and 20 days post-anthesis, respectively. The four tracks in the middle represent the four gene sets, with individual blocks showing “presence” (green) or “absence” (gray) of the gene sets in each intersection. The height of the bars in the outer layer is proportional to the intersection sizes, as indicated by the numbers on the top of the bars. The color intensity of the bars represents the *P* value significance of the intersections. (PNG 426 kb)

High resolution image (TIF 1495 kb)

Supplementary Fig. 5 GO enrichment of the genes involved in wheat grain development. **a** GO enrichment of grain-specific expression genes (SEGs). **b** GO enrichment of the differentially expressed genes in developmental grains (DEGs). The top 20 significantly enriched GO categories are shown in the histogram. The horizontal axes represent the number of enriched genes in individual GO terms. The vertical axes indicate different functional groups. (PNG 526 kb)

High resolution image (TIF 5598 kb)

Supplementary Fig. 6 KEGG enrichment of the genes involved in wheat grain development. **a** The grain-specific expression genes (SEGs). **b** The differentially expressed genes in developmental grains (DEGs). The top 20 enriched pathways are listed in bubble chart. The horizontal axes represent the enrichment factor and the vertical axes indicate pathways. The size of bubble represents the number of genes and the color of bubble represents P value. (PNG 786 kb)

High resolution image (TIF 3560 kb)

Supplementary Fig. 7 Expression trends of all genes differentially expressed during grain development. The horizontal axes indicate the grain samples of four developmental stages, GR5, GR10, GR15, and GR20 representing grain at 5, 10, 15, and 20 days post-anthesis, respectively. The vertical axes represent relative expression levels (the logarithm (log_2_) of the fold change) across wheat tissues, whereas the fold change is the ratio of the abundance of a gene in GR10, GR15, or GR20 to the abundance of the same gene in GR5. Each line in individual panel represents a gene in this profile. The horizontal axes indicate developmental grains. (PNG 1598 kb)

High resolution image (TIF 1729 kb)

Supplementary Fig. 8 GO enrichment of the genes in expression profile 15, 0, 2, and 8 shown in Fig. 3. The top 20 significantly enriched GO categories are shown in the histogram. The horizontal axes represent the number of enriched genes in individual GO term. The vertical axes indicate different functional groups. (PNG 1021 kb)

High resolution image (TIF 9116 kb)

Supplementary Fig. 9 Weighted gene co-expression network analysis (WGCNA) of developmental grain of wheat. Hierarchical cluster tree shows co-expression modules of genes in developmental grains. The modules are constructed using RNA-data from grains at 5, 10, 15, and 20 days post-anthesis, respectively. The leaves in the tree present individual genes, and the major branches are constituted of 15 modules labeled by different colors. (PNG 277 kb)

High resolution image (TIF 1852 kb)

Supplementary Fig. 10 The correlation between modules and different grain developmental stages. The scale bar in the right represents the coefficient. The closer the absolute value of the correlation between grain sample and module is, the stronger the correlation is. GR5, GR10, GR15, and GR20 represent grains at 5, 10, 15, and 20 days post-anthesis, respectively. (PNG 405 kb)

High resolution image (TIF 5884 kb)

Supplementary Fig. 11 The heatmap of the ten putative key transcription factors (TFs) and the seven predicted target genes of these TFs shown in Fig. 4. The right show gene IDs. The gene IDs with red color indicate the putative key TF genes and those with black color present the predicted gene regulated by these key TFs, the names of the genes being described within bracket. The scale bar in right indicates relative expression level of individual genes across wheat tissues. R, S, and L represent root, stem, and leaf of five-leaf stage seedlings, FL represents flag leaf of wheat plants at heading stage, YS5 represents young spike of wheat plant at early booting stage, YS15 represents spike of wheat plant at heading stage, GR5, GR10, GR15, and GR20 represent grain at 5, 10, 15, and 20 days post-anthesis, respectively. (PNG 351 kb)

High resolution image (TIF 2403 kb)

Supplementary Table 1 (XLSX 14 kb)

Supplementary Table 2 (XLSX 18 kb)

Supplementary Table 3 (XLSX 11104 kb)

Supplementary Table 4 (XLSX 10 kb)

Supplementary Table 5 (XLSX 177 kb)

Supplementary Table 6 (XLSX 3165 kb)

Supplementary Table 7 (XLSX 3400 kb)

Supplementary Table 8 (XLSX 155 kb)

Supplementary Table 9 (XLSX 186 kb)

Supplementary Table 10 (XLSX 69 kb)

Supplementary Table 11 (XLSX 896 kb)

Supplementary Table 12 (XLSX 25 kb)

Supplementary Table 13 (XLSX 16 kb)
