# Supplementary figures and images for: Global transcriptome analysis uncovers the gene co-expression regulation network and key genes involved in grain development of wheat (Triticum aestivum L.)
Source: Funct Integr Genomics. 2019 May 21;19(6):853–66. doi: 10.1007/s10142-019-00678-z (PMC6797667; doi:10.1007/s10142-019-00678-z)

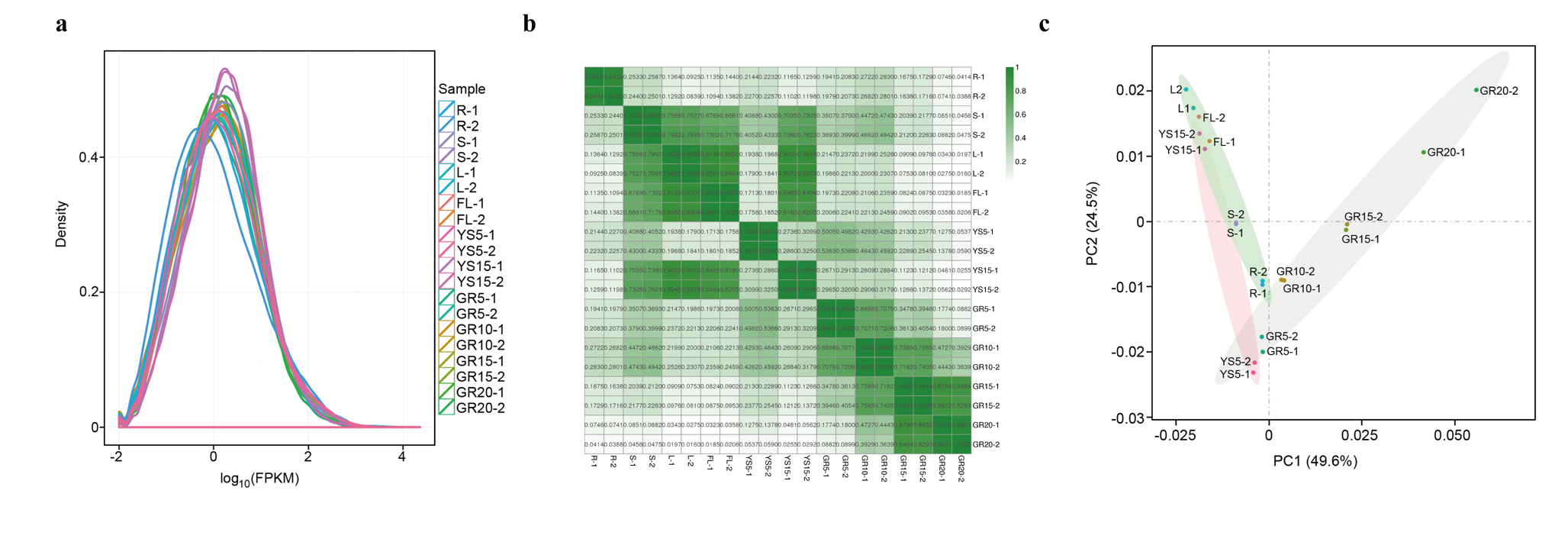

Supplement: Supplementary file 1 — Analysis of the relationship among all 20 wheat samples according to RNA-seq based gene expression values. R, S, and L represent root, stem and leaf of five-leaf stage seedling, respectively. FL represents flag leaf of wheat plant at heading stage. YS5 represents young spike of wheat at early booting stage. YS15 represents spike of wheat at heading stage. GR5, GR10, GR15, and GR20 represent grain at 5, 10, 15, and 20 days post-anthesis, respectively. a Expression density plots of all samples. FPKM, fragments per kilobase of transcript per million mapped reads. b The heatmap of sample correlation. The bar in right represents the scale of the relationship among samples, and the value in each pane represents the correlation coefficient between two samples. c Principal components analysis (PCA). All samples were clustered into three separate groups corresponding to vegetative tissues, developing spikes, and developing grains shown by green, red and gray colors, respectively. The numbers in parentheses represent the proportion of variance explained by that principal component. (PNG 455 kb) [file 10142_2019_678_Fig5_ESM.png]

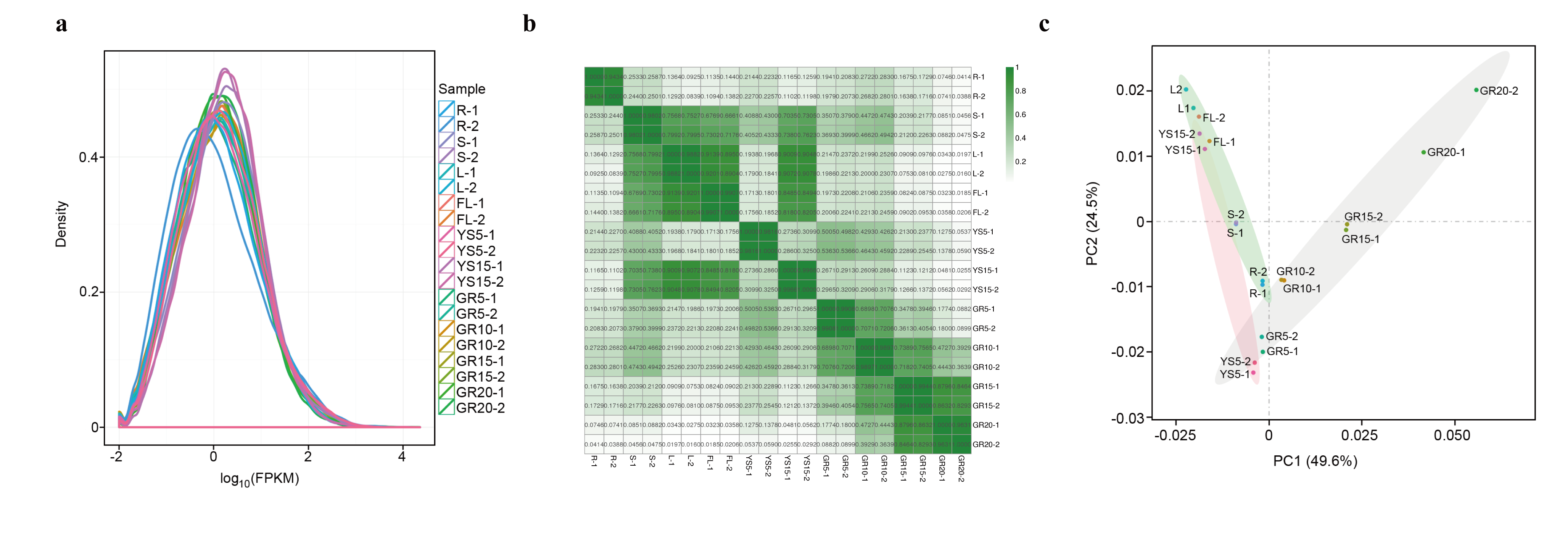

Supplement: Supplementary file 2 — High resolution image (TIF 1693 kb) [file 10142_2019_678_MOESM1_ESM.tif]

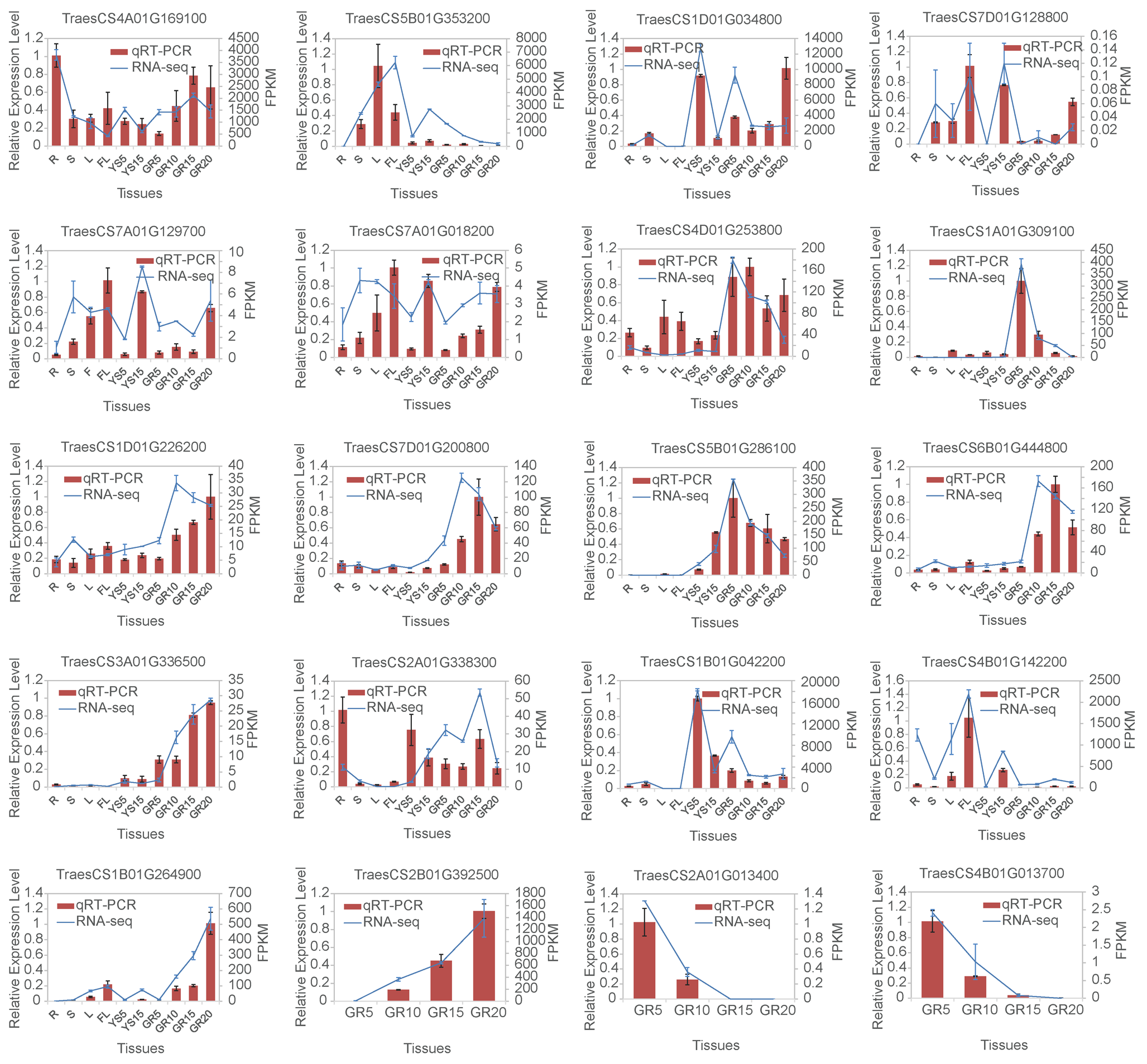

Supplement: Supplementary file 3 — Comparison of the gene expression patterns determined by quantitative real-time RT-PCR (qRT-PCR) and RNA-seq. The horizontal axis is ten wheat tissues/organs. R, S, and L represent root, stem, and leaf of five-leaf stage seedlings, respectively, FL represents flag leaf of wheat plants at heading stage, YS5 represents young spike of wheat plant at early booting stage, YS15 represents spike of wheat plant at heading stage, GR5, GR10, GR15, and GR20 represent grain at 5, 10, 15, and 20 days post-anthesis, respectively. The left vertical axes show the relative expression levels of the tested gene in individual tissues obtained by qRT-PCR and correspond to histogram, the maximum gene expression levels being defined as one. The right vertical axes are FPKM of the tested gene in individual tissues resulted from RNA-seq and correspond to line chart, whereas FPKM means fragments per kilobase of transcript per million mapped reads. The error bars on the histogram represent the standard deviation of three biological replicates, and the error bars on the line chart represent the standard deviation of two biological replicates. The annotations of these genes were listed in Supplementary Table 3. (PNG 1148 kb) [file 10142_2019_678_Fig6_ESM.png]

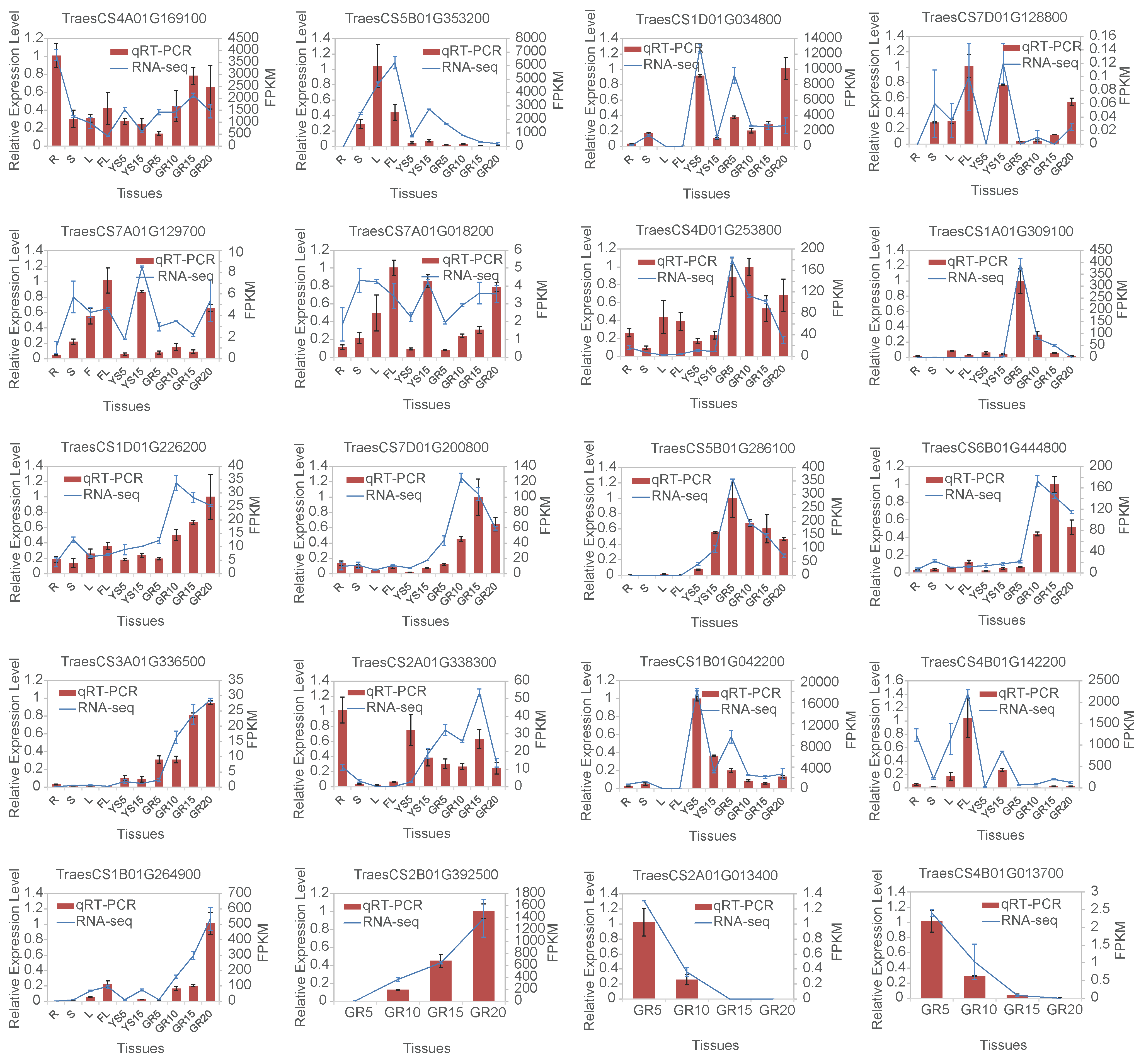

Supplement: Supplementary file 4 — High resolution image (TIF 3805 kb) [file 10142_2019_678_MOESM2_ESM.tif]

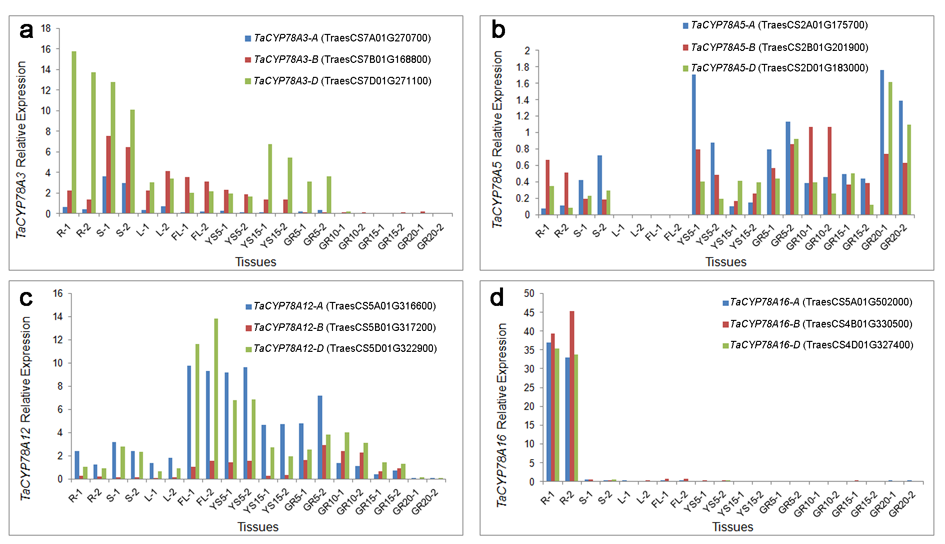

Supplement: Supplementary file 5 — Expression patterns of the homoeologous triads of TaCYP78A family members across wheat tissues/organs based on RNA-seq data. R, S, and L represent root, stem, and leaf tissue of five-leaf stage seedling, respectively. FL represents flag leaf of wheat plant at heading stage. YS5 represents young spike of wheat at early booting stage. YS15 represents spike of wheat at heading stage. GR5, GR10, GR15, and GR20 represent grain at 5, 10, 15, and 20 days post-anthesis, respectively. The number 1 or 2 the behind the names of tissue sample indicates two biological repeats, respectively. The Y axes show the relative expression levels of the tested gene in individual tissues. The numbers within brackets are the corresponding gene ID from the IWGSC RefSeq v1.0, the latest released fully annotated reference genome of bread wheat, their expression values and annotations being shown in Supplementary Table 3. (PNG 142 kb) [file 10142_2019_678_Fig7_ESM.png]

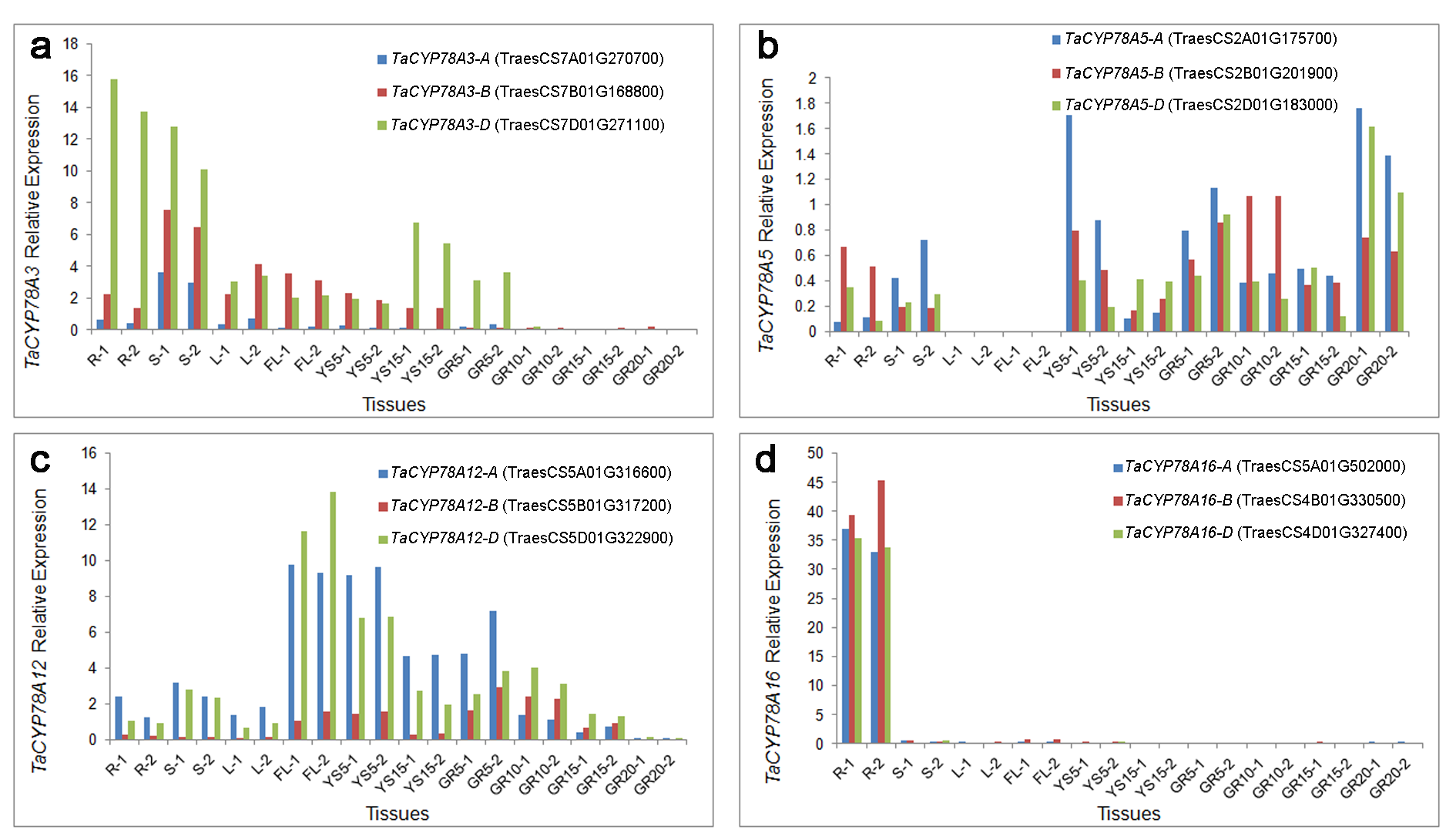

Supplement: Supplementary file 6 — High resolution image (TIF 415 kb) [file 10142_2019_678_MOESM3_ESM.tif]

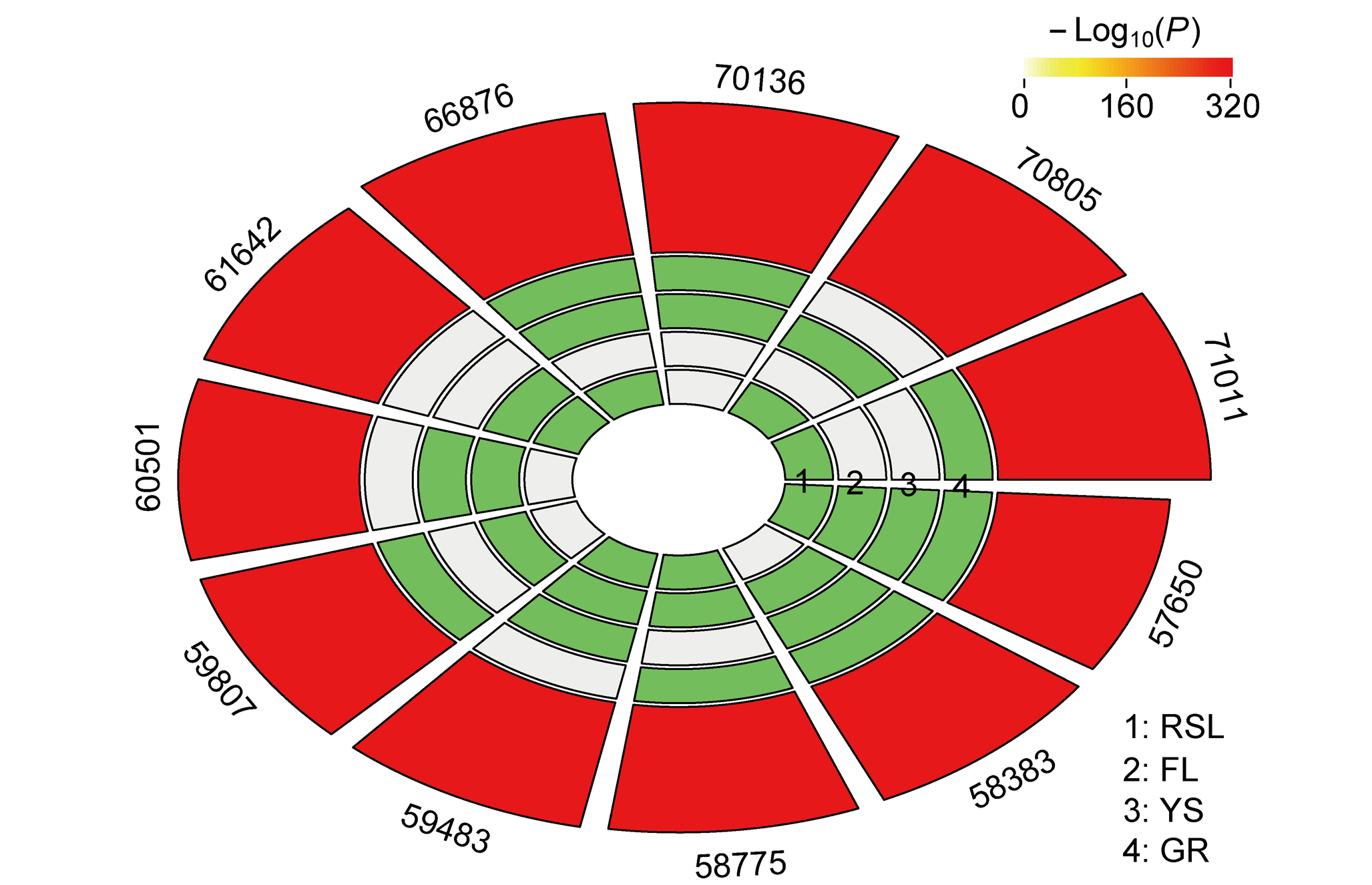

Supplement: Supplementary file 7 — Visualization of the intersections among four types of tissue sample group RSL, FL, YS, and GR. A circular plot illustrate all possible intersections and the corresponding statistics. RSL indicates the union of the genes expressed in root, stem, and leaf tissue, FL represents flag leaf of wheat plants at heading stage, YS represents the union of the genes expressed in young spike of wheat plants at early booting stage and at heading stage, GR indicates the union of the genes expressed in grains at 5, 10, 15, and 20 days post-anthesis, respectively. The four tracks in the middle represent the four gene sets, with individual blocks showing “presence” (green) or “absence” (gray) of the gene sets in each intersection. The height of the bars in the outer layer is proportional to the intersection sizes, as indicated by the numbers on the top of the bars. The color intensity of the bars represents the P value significance of the intersections. (PNG 426 kb) [file 10142_2019_678_Fig8_ESM.png]

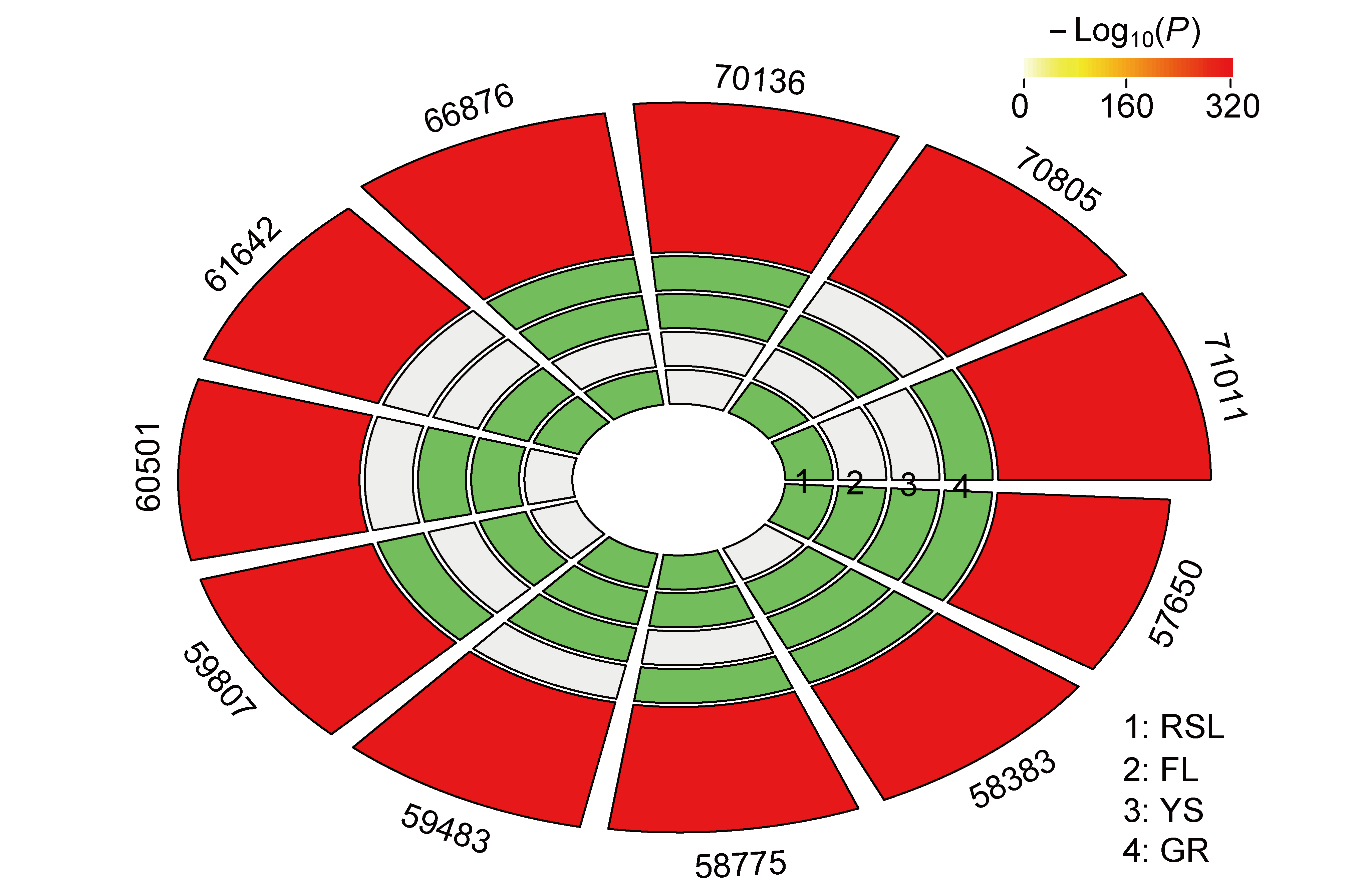

Supplement: Supplementary file 8 — High resolution image (TIF 1495 kb) [file 10142_2019_678_MOESM4_ESM.tif]

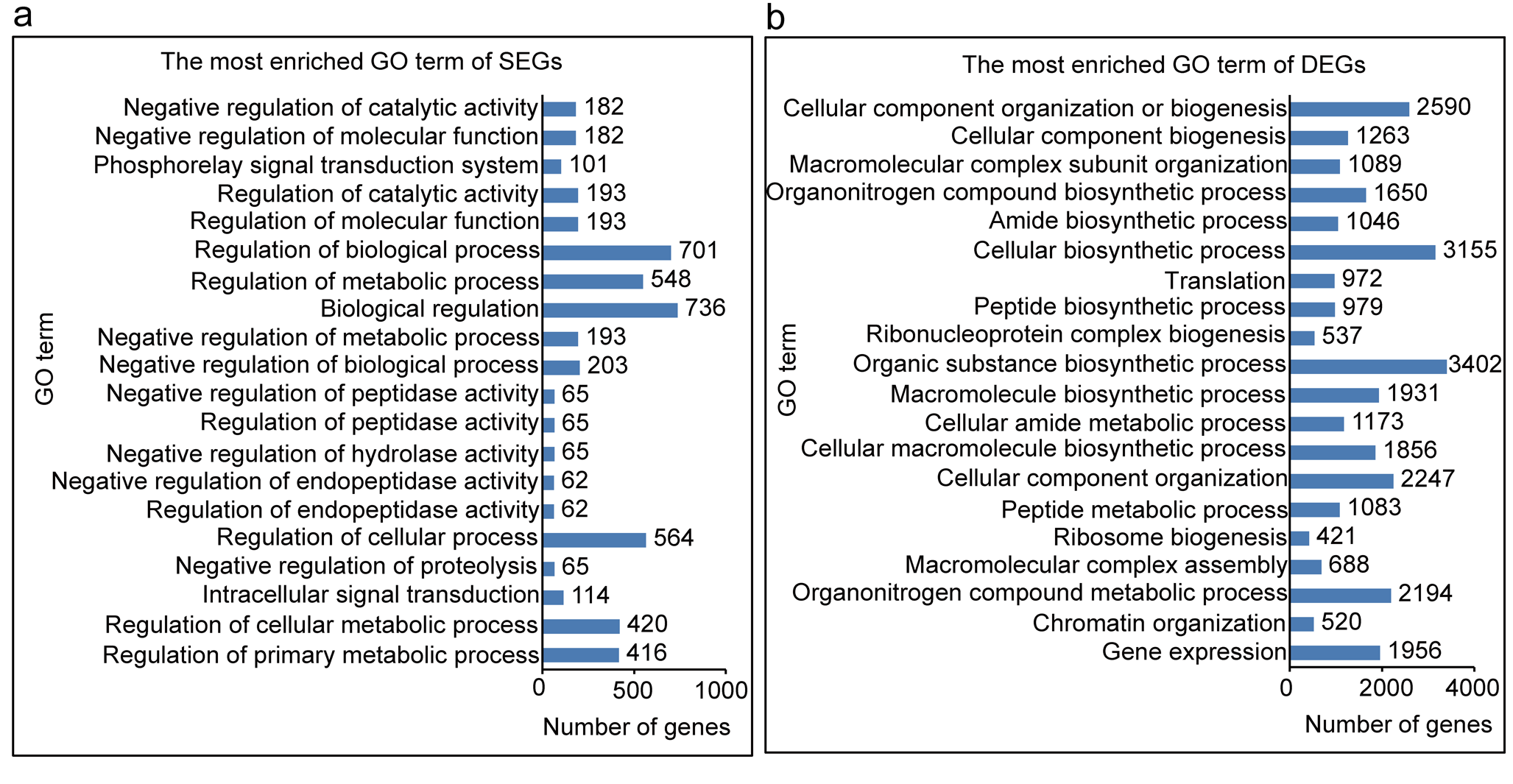

Supplement: Supplementary file 9 — GO enrichment of the genes involved in wheat grain development. a GO enrichment of grain-specific expression genes (SEGs). b GO enrichment of the differentially expressed genes in developmental grains (DEGs). The top 20 significantly enriched GO categories are shown in the histogram. The horizontal axes represent the number of enriched genes in individual GO terms. The vertical axes indicate different functional groups. (PNG 526 kb) [file 10142_2019_678_Fig9_ESM.png]

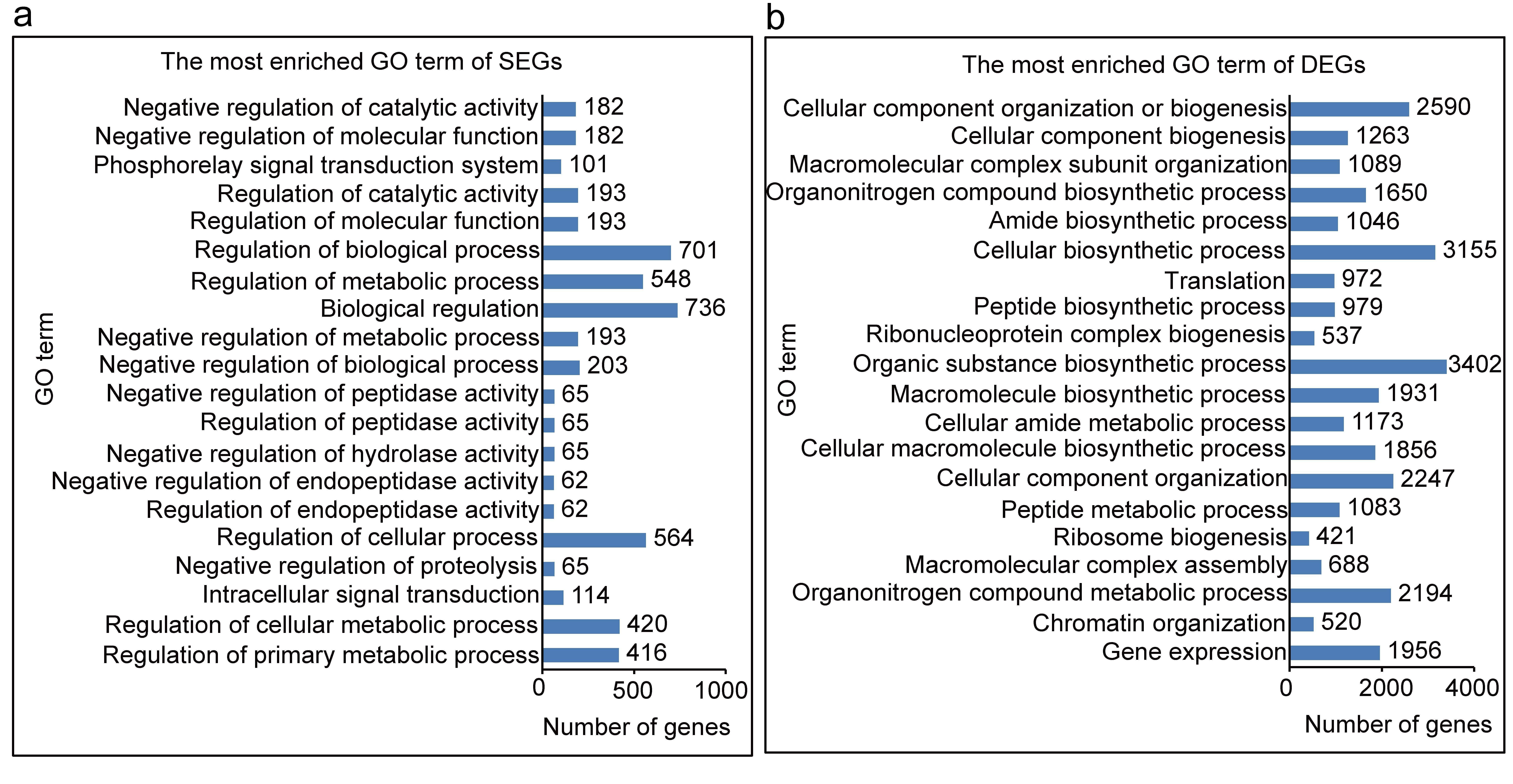

Supplement: Supplementary file 10 — High resolution image (TIF 5598 kb) [file 10142_2019_678_MOESM5_ESM.tif]

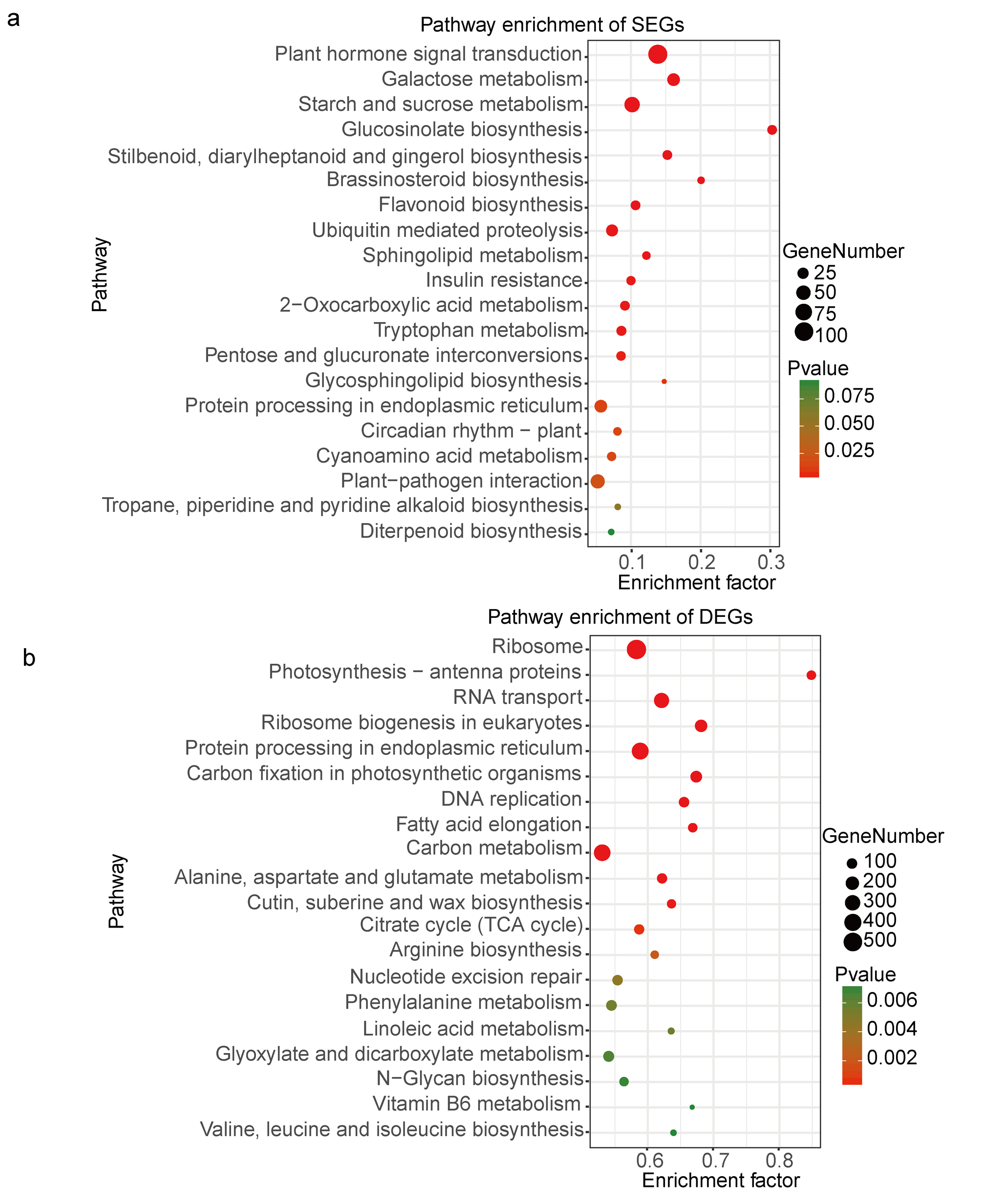

Supplement: Supplementary file 11 — KEGG enrichment of the genes involved in wheat grain development. a The grain-specific expression genes (SEGs). b The differentially expressed genes in developmental grains (DEGs). The top 20 enriched pathways are listed in bubble chart. The horizontal axes represent the enrichment factor and the vertical axes indicate pathways. The size of bubble represents the number of genes and the color of bubble represents P value. (PNG 786 kb) [file 10142_2019_678_Fig10_ESM.png]

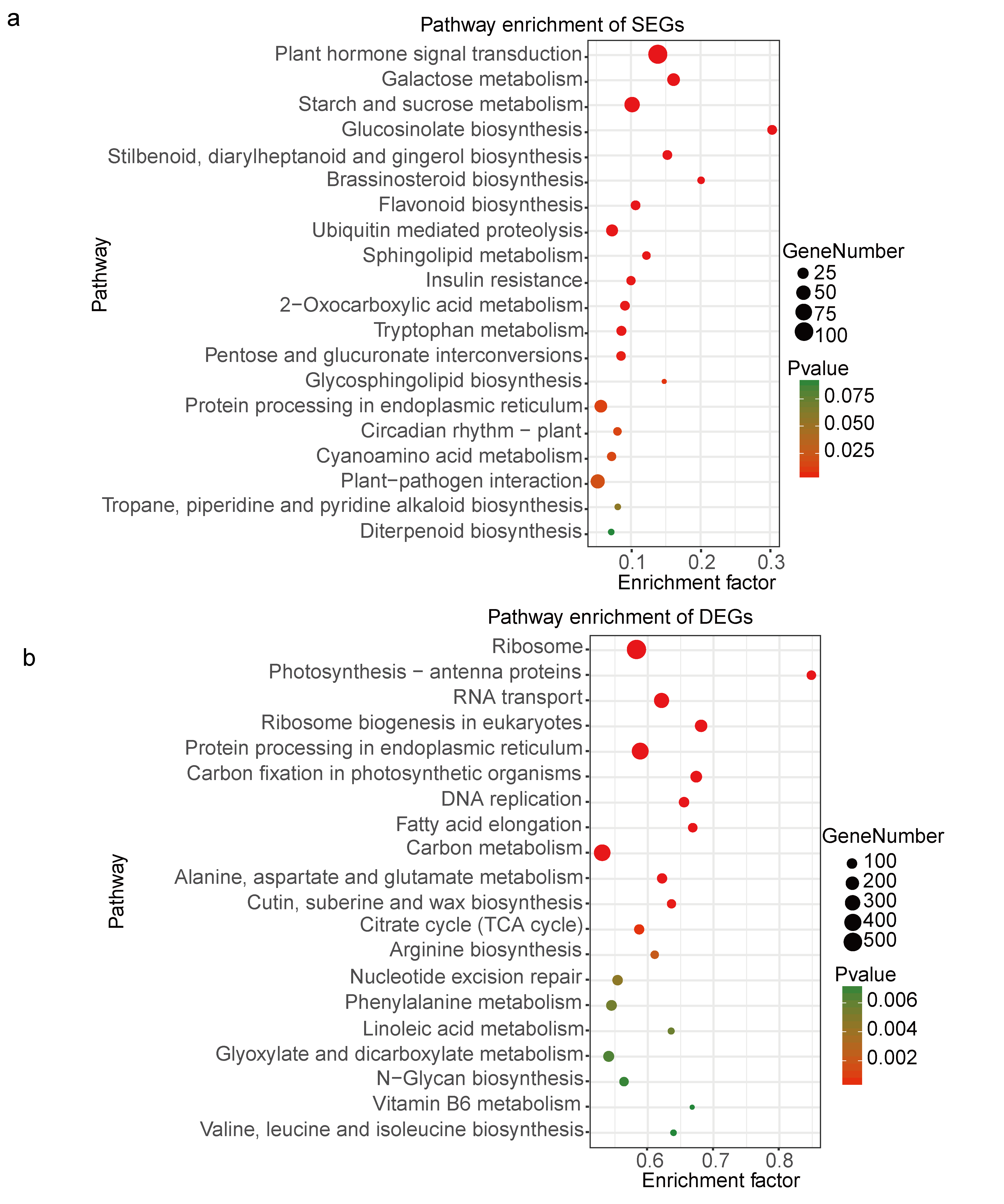

Supplement: Supplementary file 12 — High resolution image (TIF 3560 kb) [file 10142_2019_678_MOESM6_ESM.tif]

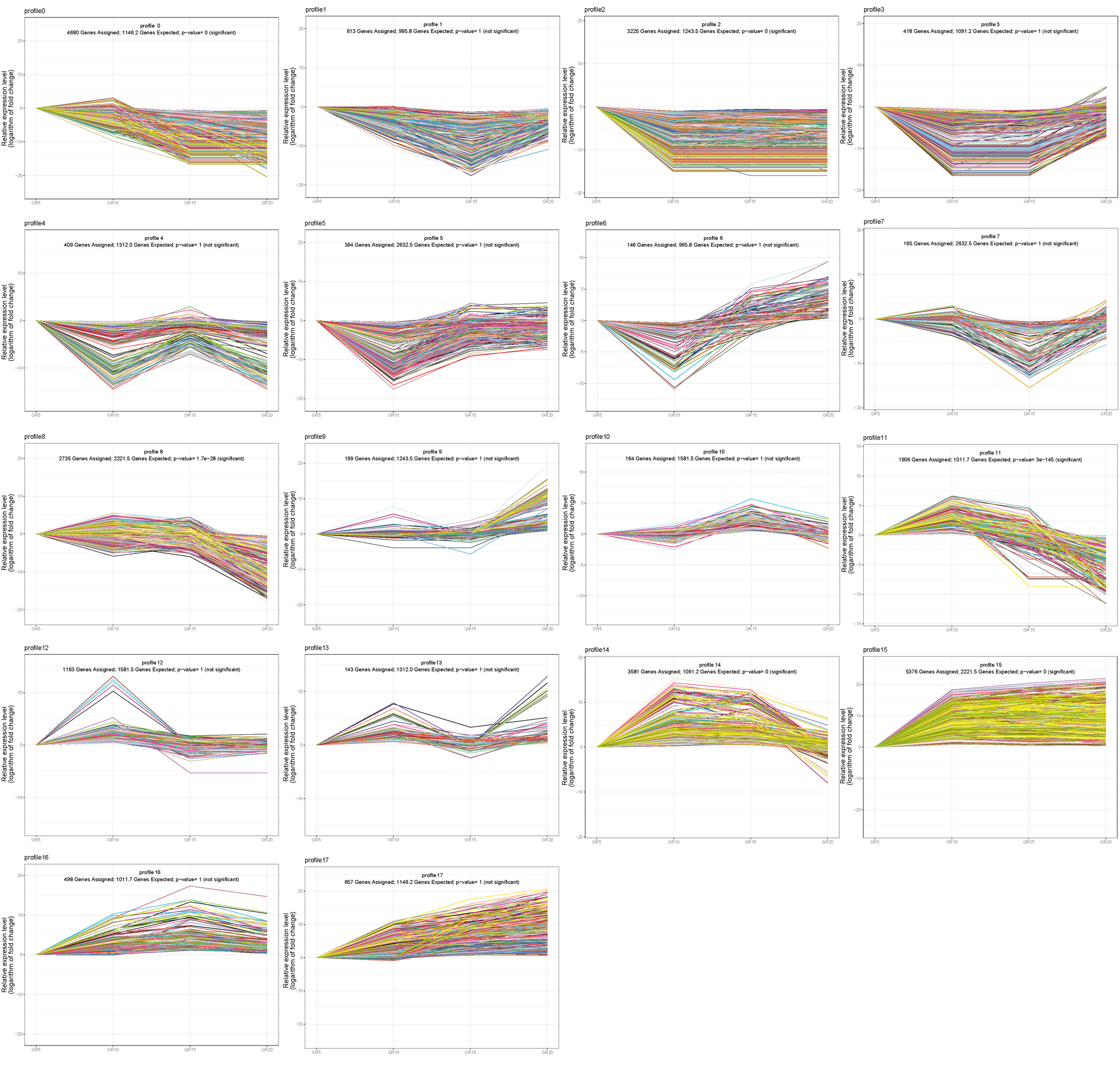

Supplement: Supplementary file 13 — Expression trends of all genes differentially expressed during grain development. The horizontal axes indicate the grain samples of four developmental stages, GR5, GR10, GR15, and GR20 representing grain at 5, 10, 15, and 20 days post-anthesis, respectively. The vertical axes represent relative expression levels (the logarithm (log2) of the fold change) across wheat tissues, whereas the fold change is the ratio of the abundance of a gene in GR10, GR15, or GR20 to the abundance of the same gene in GR5. Each line in individual panel represents a gene in this profile. The horizontal axes indicate developmental grains. (PNG 1598 kb) [file 10142_2019_678_Fig11_ESM.png]

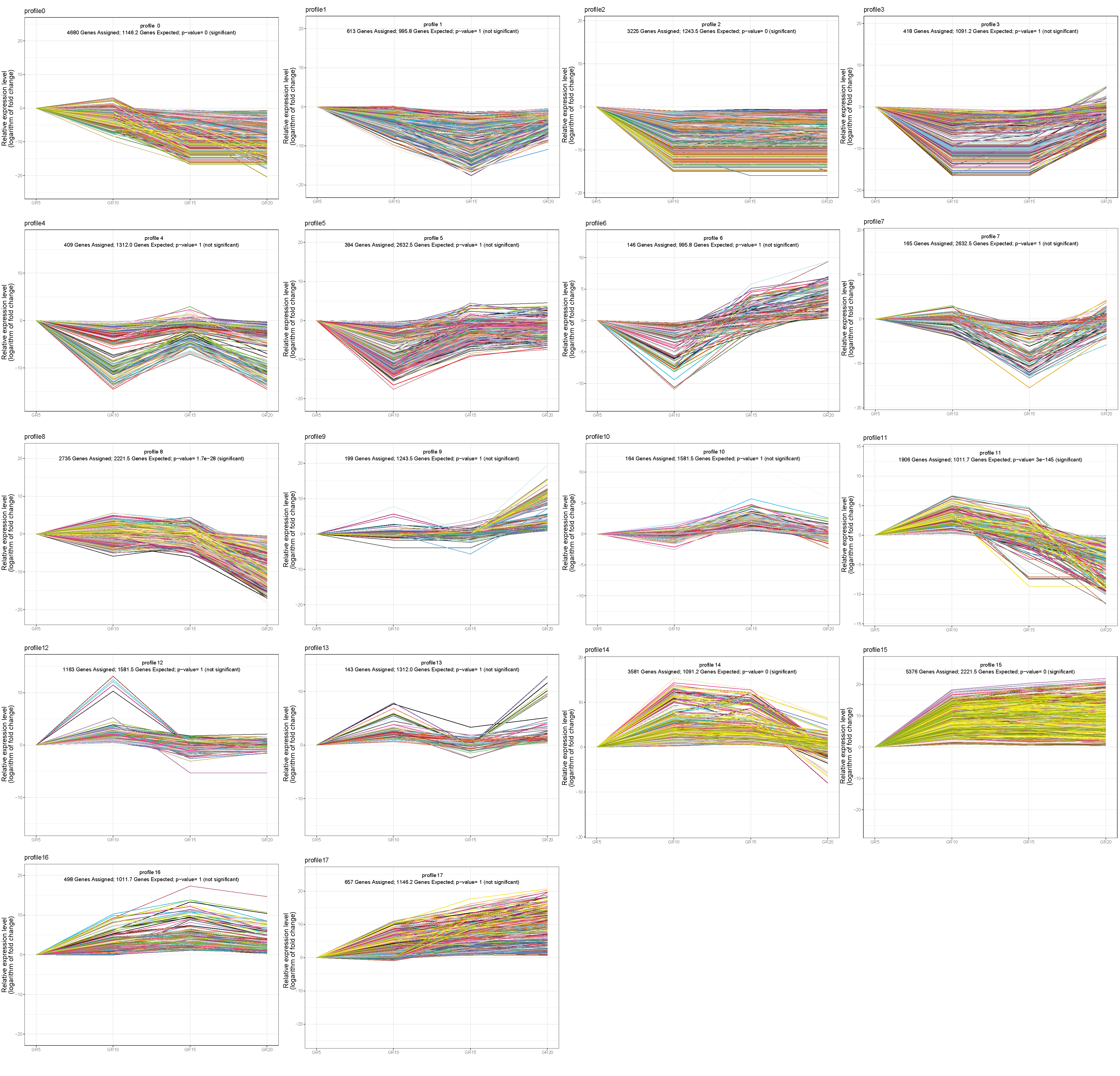

Supplement: Supplementary file 14 — High resolution image (TIF 1729 kb) [file 10142_2019_678_MOESM7_ESM.tif]

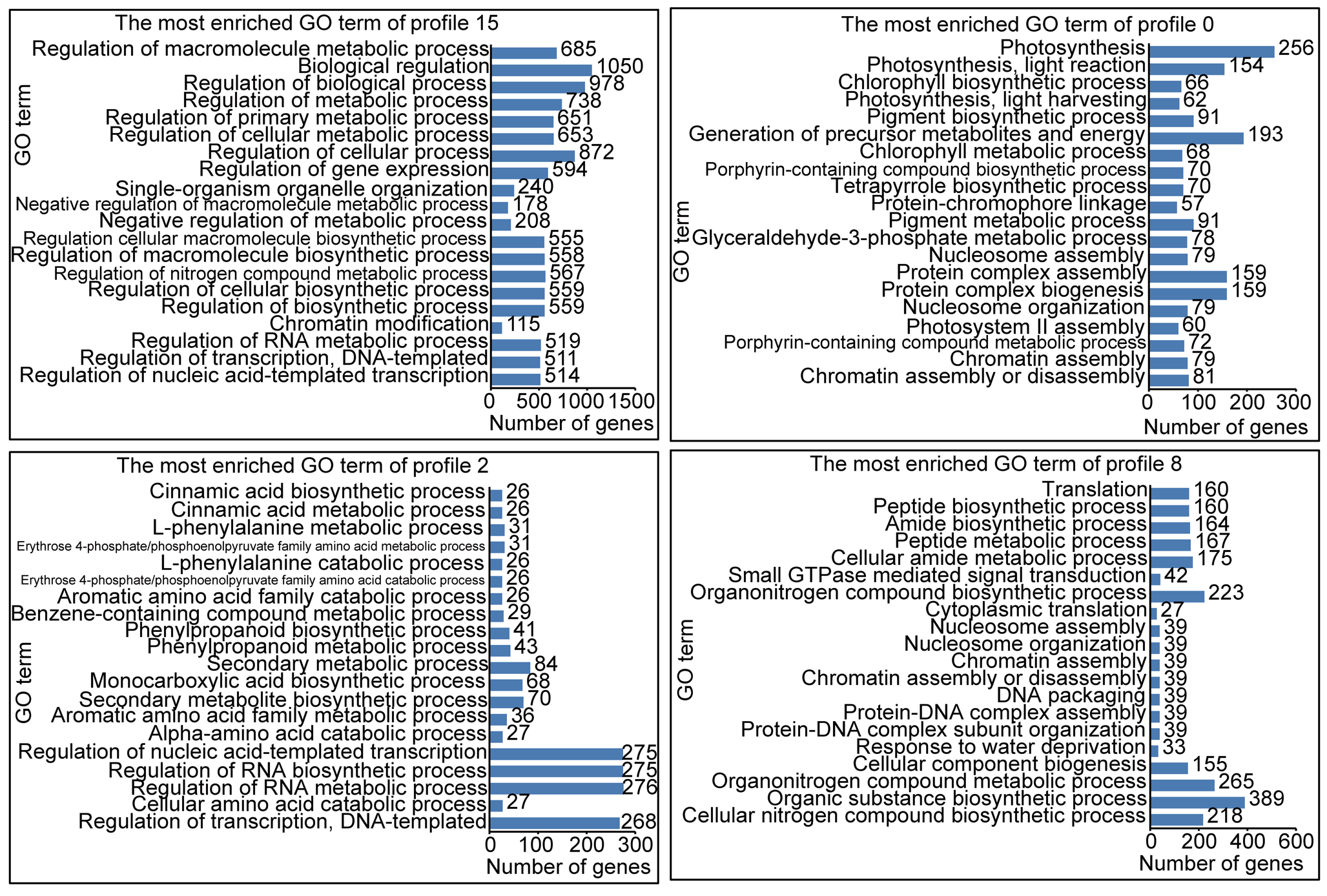

Supplement: Supplementary file 15 — GO enrichment of the genes in expression profile 15, 0, 2, and 8 shown in Fig. 3. The top 20 significantly enriched GO categories are shown in the histogram. The horizontal axes represent the number of enriched genes in individual GO term. The vertical axes indicate different functional groups. (PNG 1021 kb) [file 10142_2019_678_Fig12_ESM.png]

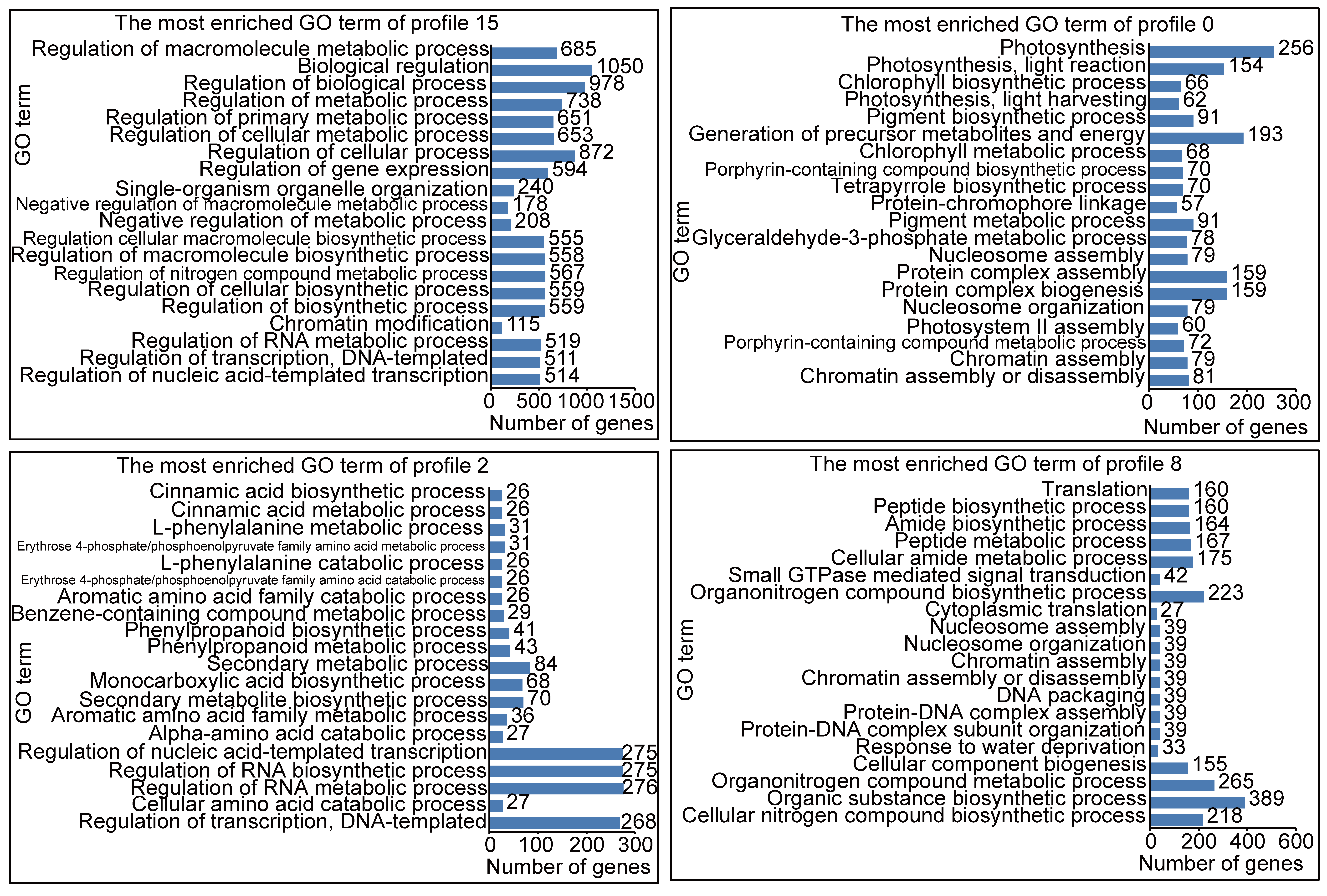

Supplement: Supplementary file 16 — High resolution image (TIF 9116 kb) [file 10142_2019_678_MOESM8_ESM.tif]

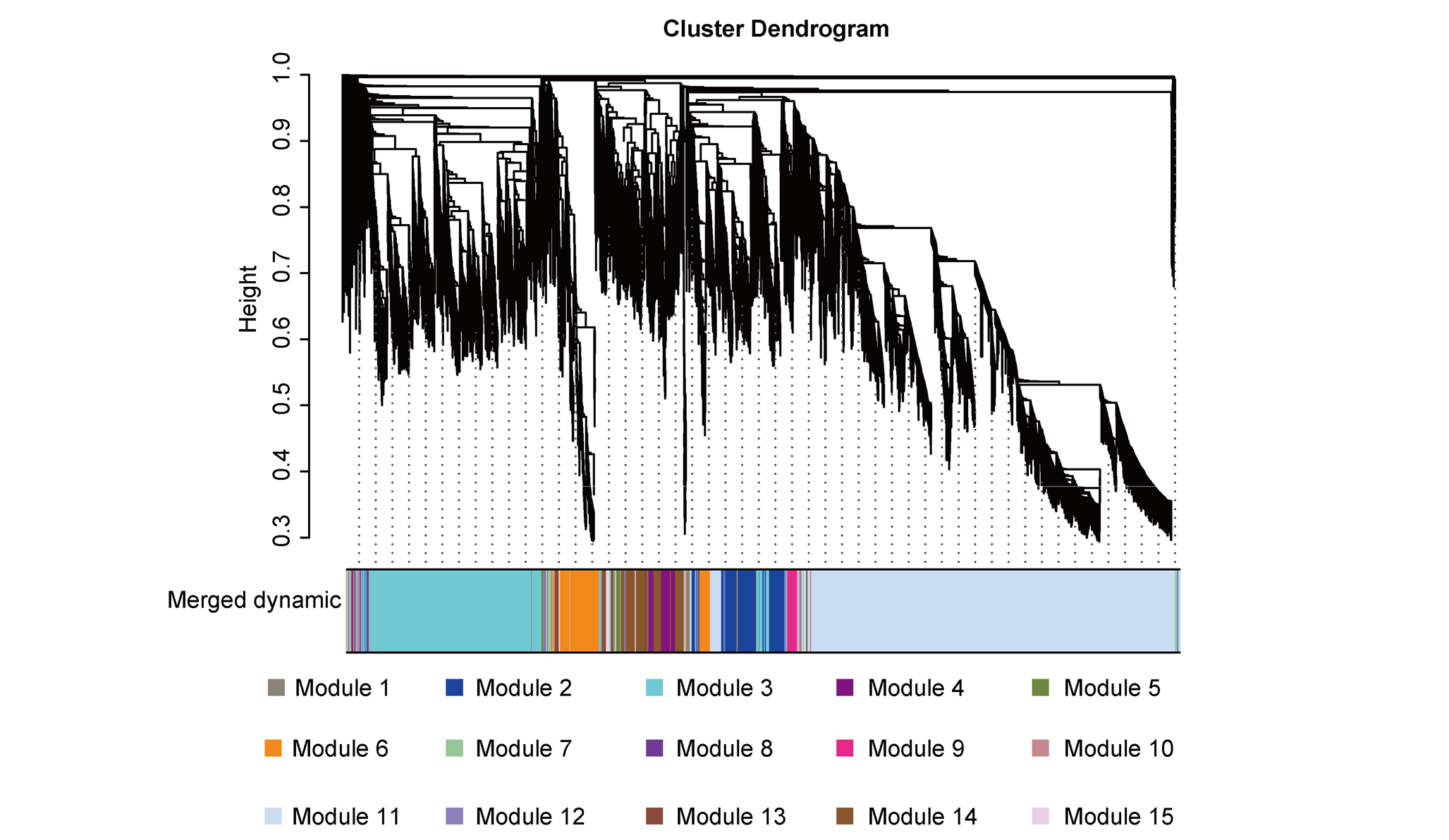

Supplement: Supplementary file 17 — Weighted gene co-expression network analysis (WGCNA) of developmental grain of wheat. Hierarchical cluster tree shows co-expression modules of genes in developmental grains. The modules are constructed using RNA-data from grains at 5, 10, 15, and 20 days post-anthesis, respectively. The leaves in the tree present individual genes, and the major branches are constituted of 15 modules labeled by different colors. (PNG 277 kb) [file 10142_2019_678_Fig13_ESM.png]

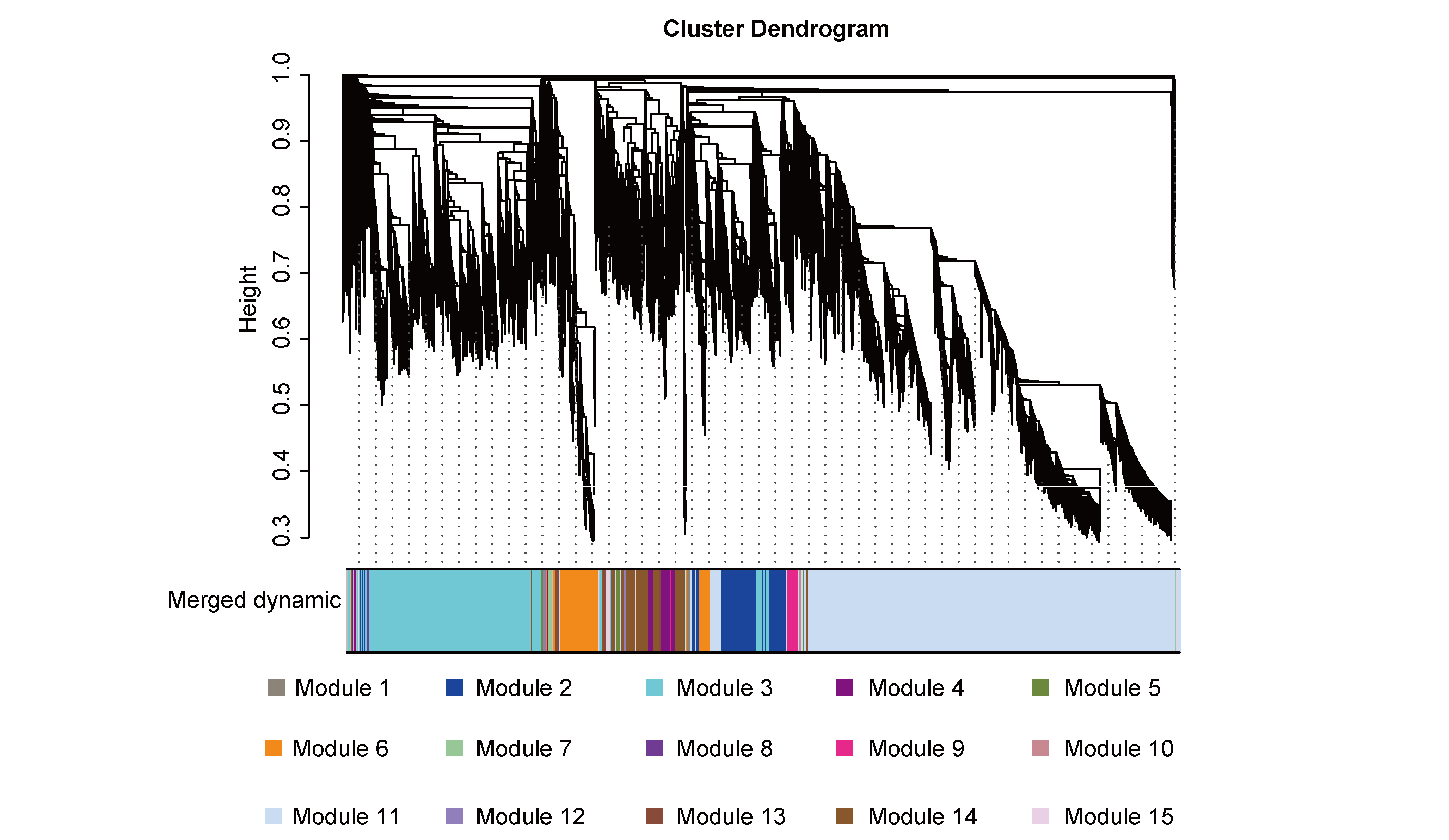

Supplement: Supplementary file 18 — High resolution image (TIF 1852 kb) [file 10142_2019_678_MOESM9_ESM.tif]

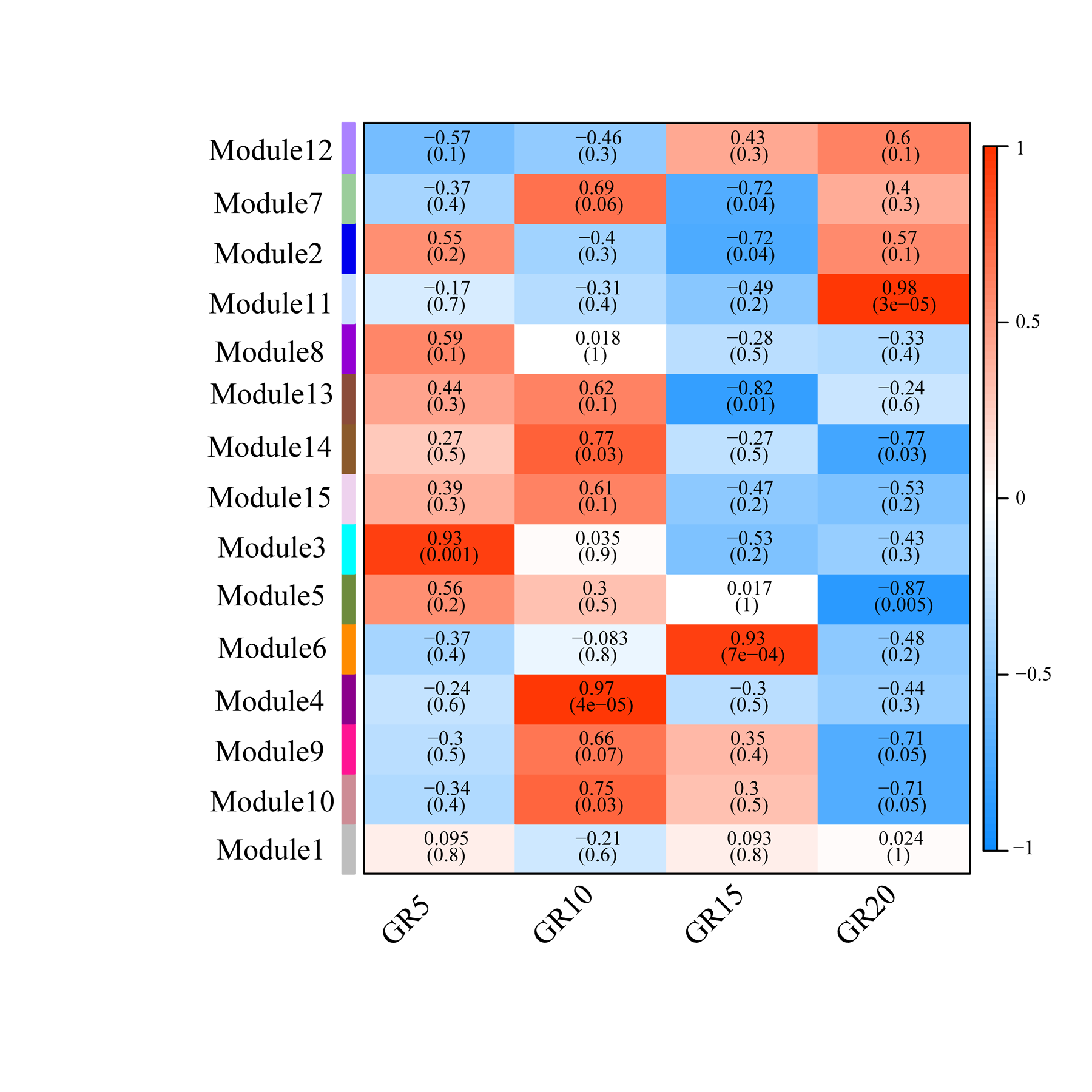

Supplement: Supplementary file 19 — The correlation between modules and different grain developmental stages. The scale bar in the right represents the coefficient. The closer the absolute value of the correlation between grain sample and module is, the stronger the correlation is. GR5, GR10, GR15, and GR20 represent grains at 5, 10, 15, and 20 days post-anthesis, respectively. (PNG 405 kb) [file 10142_2019_678_Fig14_ESM.png]

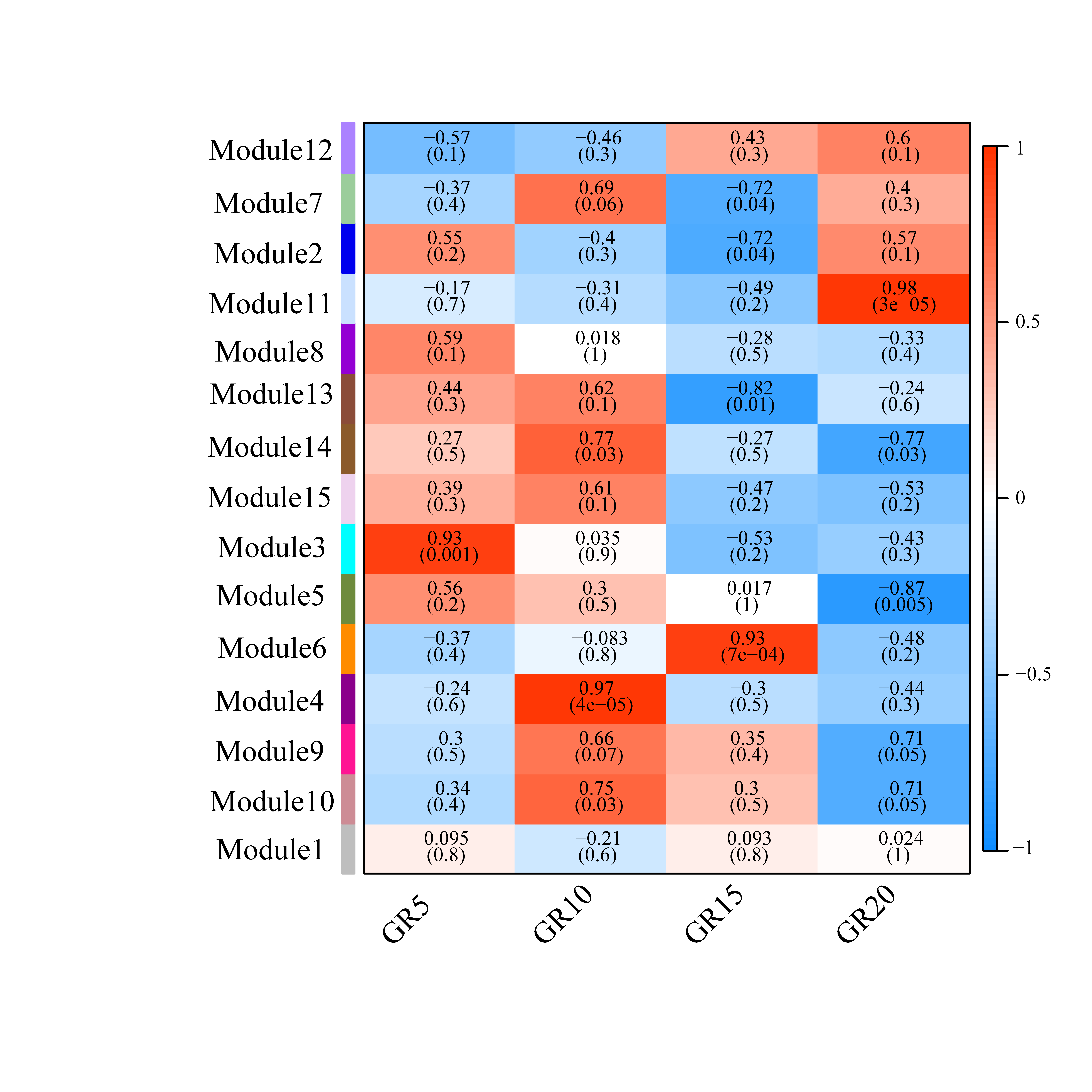

Supplement: Supplementary file 20 — High resolution image (TIF 5884 kb) [file 10142_2019_678_MOESM10_ESM.tif]

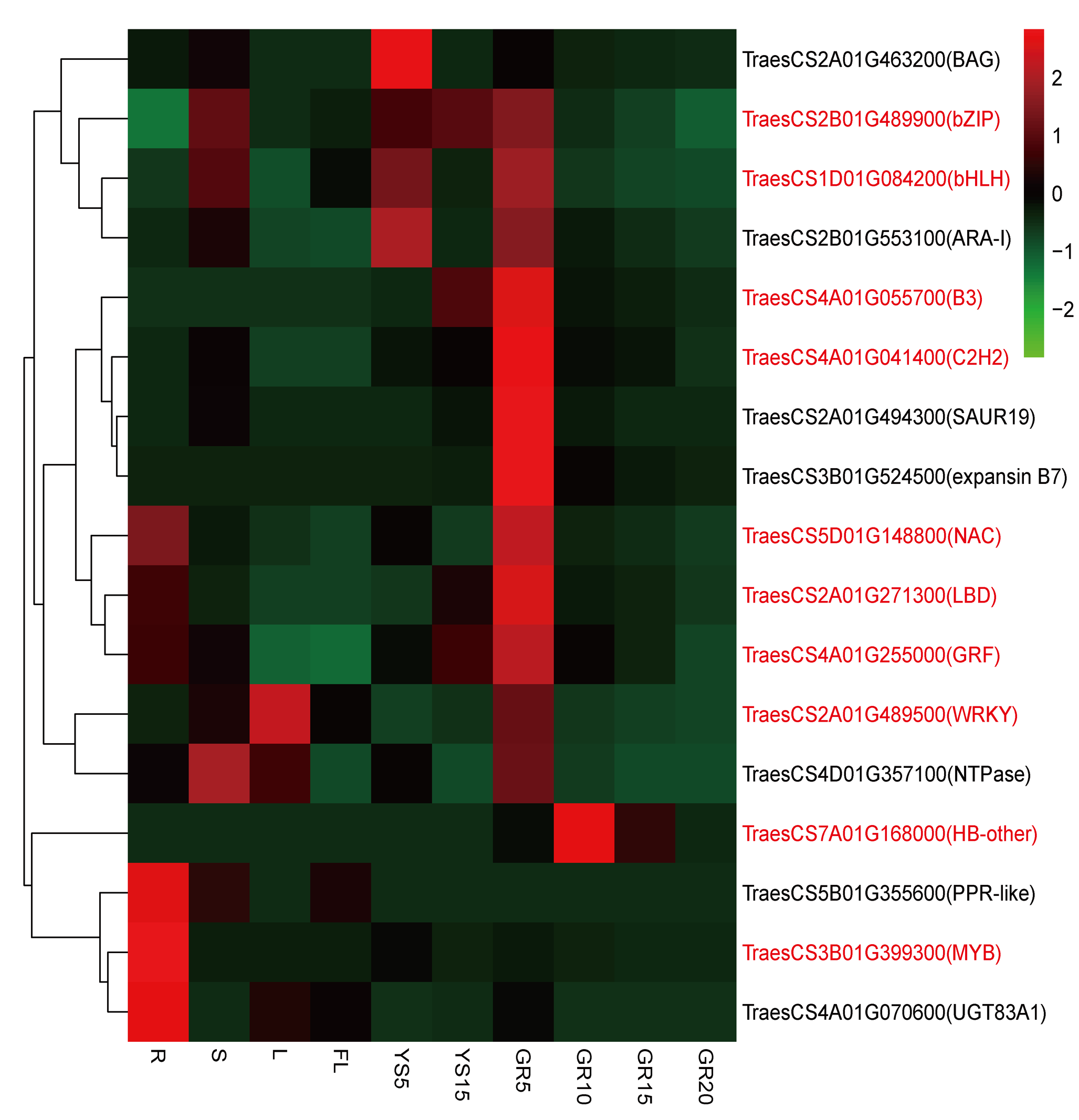

Supplement: Supplementary file 21 — The heatmap of the ten putative key transcription factors (TFs) and the seven predicted target genes of these TFs shown in Fig. 4. The right show gene IDs. The gene IDs with red color indicate the putative key TF genes and those with black color present the predicted gene regulated by these key TFs, the names of the genes being described within bracket. The scale bar in right indicates relative expression level of individual genes across wheat tissues. R, S, and L represent root, stem, and leaf of five-leaf stage seedlings, FL represents flag leaf of wheat plants at heading stage, YS5 represents young spike of wheat plant at early booting stage, YS15 represents spike of wheat plant at heading stage, GR5, GR10, GR15, and GR20 represent grain at 5, 10, 15, and 20 days post-anthesis, respectively. (PNG 351 kb) [file 10142_2019_678_Fig15_ESM.png]

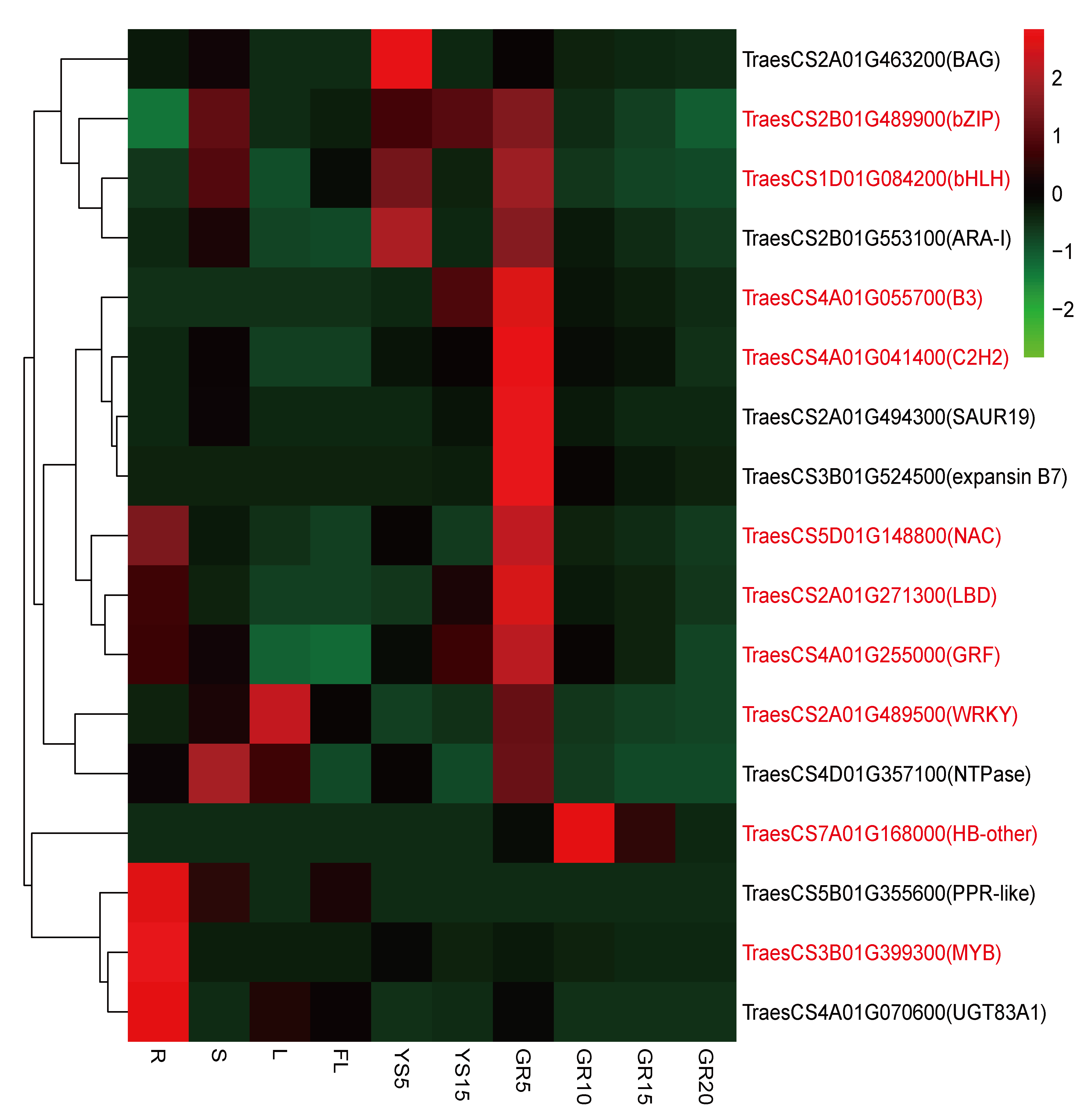

Supplement: Supplementary file 22 — High resolution image (TIF 2403 kb) [file 10142_2019_678_MOESM11_ESM.tif]
